# Scientific contributions of Alexandr P. Rasnitsyn, 1959 to present

**DOI:** 10.3897/zookeys.130.1917

**Published:** 2011-09-24

**Authors:** Michael S. Engel, Dmitry E. Shcherbakov

**Affiliations:** 1 Division of Entomology (Paleoentomology), Natural History Museum, and Department of Ecology & Evolutionary Biology, 1501 Crestline Drive – Suite 140, University of Kansas, Lawrence, Kansas 66045, USA; 2 Borissiak Paleontological Institute, Russian Academy of Sciences, Profsoyuznaya ul. 123, 117997 Moscow, Russia

## Introduction and format

The following list provides citations for 363 scientific contributions, including 13 edited volumes and books, produced by Alexandr P. Rasnitsyn. Naturally, the list presented here is a static representation of Alex’s contributions and we look forward to years of his forthcoming publications to our shared science. Thus, this list only encompasses those papers published as of 1 August 2011. We have published it here so as to bring to the attention of colleagues Alex’s numerous and multifaceted accomplishments, and why he so richly deserves our admiration. In addition, we believe the following list is the most thorough and accurate accounting of his published scientific activities. Given that many of his works are in his native Russian, we believe this list will serve as a tool for directing interested individuals to English translations, where available.

In elaborating this list we have taken a detailed approach to cataloguing Alex’s numerous publications, adopting the philosophy that bibliography is a science of its own and provides an accurate description of a work or contribution to a work (e.g., Gaskell 1972). It is with this philosophy in mind that the list was constructed and how to determine what should, or should not, constitute an individual citation, the appropriate form of citation, etc.

To the best of our ability we have checked article and serial titles, as well as dates of publication and complete citations against original sources, either from the original series or official reprints. In addition, some serials and edited volumes have been checked against library copies at the University of Kansas (Lawrence) or the Library of Congress (Washington DC). Several of Alex’s contributions appear in special edited issues of otherwise serial publications. These are cited as they appear in the journal series, with the editors and titles of the special issues following in parentheticals.

## Serial titles

Understandably many of the serials in which Alex has published have titles in Cyrillic. The transliterated versions of these are used throughout the bibliography and we have provided here a summary of those titles and their English equivalents.

*Byulleten’ Moskovskogo Obshchestva Ispytateley Prirody, Otdel Biologicheskiy* [Bulletin of the Moscow Society of Naturalists, Series Biology]

*Byulleten’ Moskovskogo Obshchestva Ispytateley Prirody, Otdel Geologicheskiy* [Bulletin of the Moscow Society of Naturalists, Series Geology]

*Doklady* *Akademii Nauk SSSR* (since 1992, *Doklady* *Akademii Nauk*)[Transactions of the Academy of Sciences, USSR (since 1992, of the Russian Academy of Sciences), or sometimes appearing simply as “Doklady” followed by the English name of the particular section, e.g., “Doklady. Biological Sciences Section”]

*Doklady Moskovskogo Obshchestva Ispytatelei Prirody, Zoologia i Botanika* [Reports of the Moscow Society of Naturalists, Zoology and Botany]

*Entomologicheskoe Obozrenie* [Entomological Review]

*Izvestiya Akademii Nauk, Seriya Biologicheskaya* [Bulletin of the Academy of Sciences, Biology Series]

*Paleontologicheskiy Zhurnal* [Paleontological Journal]

*Trudy Paleontologicheskogo Instituta Akademii Nauk SSSR* (since 1992, …*Rossiyskoy Akademii Nauk*)[Transactions of the Paleontological Institute, Academy of Sciences, USSR (since 1992, Russian Academy of Sciences)]

*Trudy Russkogo Entomologicheskogo Obshchestva* [Proceedings of the Russian Entomological Society]

*Trudy Sovmestnoy Sovetsko-Mongol’skoy Paleontologicheskoy Ekspeditsii* [Transactions of the Joint Soviet-Mongolian Paleontological Expedition]

*Uspekhi Sovremennoy Biologii* [Achievements in Modern Biology]

*Vestnik Permskogo Universiteta, Geologiya* [Bulletin of Perm’ University, Geology]

*Vestnik Zoologii* [Zoological Herald]

*Zhurnal Obshchey Biologii* [Journal of General Biology]

*Zoologicheskiy Zhurnal* [Zoological Journal]

**Figure:**
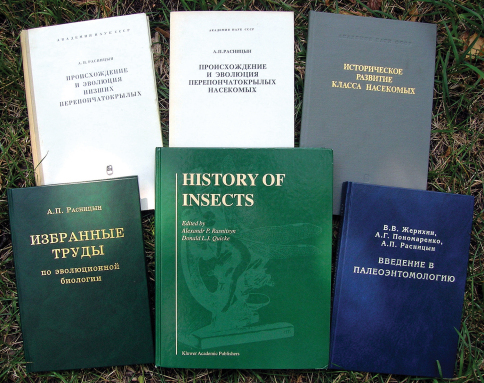


## Publications of Alexandr P. Rasnitsyn

1 January 1959–1 August 2011

1. Rasnitsyn AP (1959) Hibernation sites of ichneumon flies (Hymenoptera, Ichneumonidae). Entomologicheskoe Obozrenie 38(3): 546–553 [In Russian, with English translation in Entomological Review, 1960, 38(3): 491–498].

2. Rasnitsyn AP (1963) Late Jurassic Hymenoptera of Karatau. Paleontologicheskiy Zhurnal 1963(1): 86–99 [In Russian].

3. Rasnitsyn AP (1963) Observation on the freezing of insects. Zoologicheskiy Zhurnal 42(7): 1104–1105 [In Russian, with English summary].

4. Rasnitsyn AP (1964) On hibernation of ichneumon-flies (Hymenoptera, Ichneumonidae). Entomologicheskoe Obozrenie 43(1): 46–51 [In Russian, with English translation in Entomological Review, 1964, 38(3): 491–498].

5. Rasnitsyn AP (1964) New Triassic Hymenoptera of the middle Asia. Paleontologicheskiy Zhurnal 1964(1): 88–96 [In Russian].

6. Rasnitsyn AP (1965) Certain aspects of the interrelations between morphogenesis and growth in the evolution of insect ontogeny. Entomologicheskoe Obozrenie 44(3): 476–485 [In Russian, with English translation in Entomological Review, 1965, 44: 279–284].

7. Rasnitsyn AP (1965) Notes on the biology, systematics, and phylogeny of Xyelinae (Hymenoptera, Xyelidae). Polskie Pismo Entomologiczne 35(12): 483–519 [In Russian, with English keys and summary].

8. Rasnitsyn AP (1966) New Xyelidae (Hymenoptera) from the Mesozoic of Asia. Paleontologicheskiy Zhurnal 1966(4): 69–85 [In Russian, with English translation in International Geology Review, 1967, 9(5): 723–737].

9. Rasnitsyn AP (1966) The key of superfamilies and families of Hymenoptera. Entomologicheskoe Obozrenie 45(3): 599–611 [In Russian, with English translation in Entomological Review, 1966, 45(3): 340–347].

10. Ponomarenko AG, Rasnitsyn AP (1966) Phylogenetic and morphofunctional criteria of similarity and disparity in taxonomic practice. Byulleten’ Moskovskogo Obshchestva Ispytateley Prirody, Otdel Biologicheskiy 71(3): 146 [In Russian].

11. Rasnitsyn AP (1966) Reinforcement of regulator as a criterion of the evolutionary progress. Byulleten’ Moskovskogo Obshchestva Ispytateley Prirody, Otdel Biologicheskiy 71(3): 149–150 [In Russian].

12. Rasnitsyn AP (1966) On the failure of Dollo’s law in the evolution of Symphyta (Insecta, Hymenoptera). Byulleten’ Moskovskogo Obshchestva Ispytateley Prirody, Otdel Geologicheskiy 39(6): 139–140 [In Russian].

13. Rasnitsyn AP (1967) An attempt of the causal analysis of the process of oligomerization of the homological organs. Byulleten’ Moskovskogo Obshchestva Ispytateley Prirody, Otdel Biologicheskiy 72(4): 150 [In Russian].

14. Ponomarenko AG, Rasnitsyn AP (1967) On a method of the tentative estimation of diversity in past local faunas. Paleontologicheskiy Zhurnal 1967(3): 98–105 [In Russian].

15. Rasnitsyn AP (1968) New Mesozoic sawflies (Hymenoptera, Symphyta). In: Rohdendorf BB (Ed) Jurassic Insects of Karatau. Nauka Press, Moscow, 190–236 [total pages 252 pp., +25 pls.] [In Russian].

16. Rasnitsyn AP (1968) On the evolution of the function of ovipositor in relation to the origin of parasitism in Hymenoptera. Entomologicheskoe Obozrenie 47(1): 61–70 [In Russian, with English translation in Entomological Review 47: 35–50].

17. Rasnitsyn AP (1969) Origin and evolution of lower Hymenoptera. Trudy Paleontologicheskogo Instituta Akademii Nauk SSSR 123: 1–196 [In Russian, with English translation by Amerind Co., New Delhi, 1979].

18. Kisseleva KV, Razumovsky SM, Rasnitsyn AP (1969) Boundaries between plant communities and dynamics of vegetation. Zhurnal Obshchey Biologii 30(2): 123–131 [In Russian, with English summary].

19. Kisseleva KV, Razumovsky SM, Rasnitsyn AP (1969) On the spatio-temporal organisation of the biocenosis. Doklady Moskovskogo Obshchestva Ispytateley Prirody, Zoologia i Botanika 1967: 92–93 [In Russian].

20. Rasnitsyn AP (1969) On the cause of some kinds of orthogenesis. Doklady Moskovskogo Obshchestva Ispytateley Prirody, Zoologia i Botanika 1967: 90–91 [In Russian].

21. Rasnitsyn AP (1969) On the causes of morpho-functional progress. In: Problems of the Progressive Development in the Living World and in Technology. Abstracts of Symposium 28–30 October 1969. Institute for the history of science and technology, Leningrad, 94–98 [In Russian].

22. Rasnitsyn AP (1971) Evolution of Xyelidae (Hymenoptera). Trudy Paleontologicheskogo Instituta Akademii Nauk SSSR 130: 187–196 [In Russian]. [Obruchev DV, Shimansky VN (Eds) Current problems of palaeontology. Trudy Paleontologicheskogo Instituta Akademii Nauk SSSR, 1971, 130: 1–380].

23. Rasnitsyn AP (1971) A contribution to the causes of morpho-functional progress. Zhurnal Obshchey Biologii 32(5): 549–556 [In Russian, with English summary].

24. Ponomarenko AG, Rasnitsyn AP (1971) On phenetic and phylogenetic systems. Zoologicheskiy Zhurnal 50(1): 5–14 [In Russian, with English summary].

25. Rasnitsyn AP (1971) On the origin of Hymenoptera. In: Proceedings of XIII International Congress of Entomology [Volume 1]. Nauka Press, Leningrad, 289 pp.

26. Rasnitsyn AP (1971) On irreducibility of the macroevolutionary processes to the microevolution. In: Philosophical Problems of Evolutionary Theory (Materials of Symposium) [Volume 2]. Nauka Press, Moscow, 171–178. [In Russian].

27. Rasnitsyn AP (1971) On some taxometric methods of analysis of the system of organisms. In: Abstracts of the All-Union Colloquium on Mathematical Methods in Paleontology. Institute of Geology, Academy of Sciences, Estonian SSR, Aegviidu (Estonia), 42–45. [In Russian].

28. Rasnitsyn AP, Khovanov GM (1971) A more precise method of the diversity estimation of local faunas. In: Abstracts of the All-Union Colloquium on Mathematical Methods in Paleontology. Institute of Geology, Academy of Sciences, Estonian SSR, Aegviidu (Estonia), 45–48 [In Russian].

29. Ponomarenko AG, Rasnitsyn AP (1971) Symposium on the philosophic problems of evolutionary theory. Paleontologicheskiy Zhurnal 1971(3): 130–131 [In Russian].

30. Rasnitsyn AP (1971) On some mechanisms of speciation. Doklady Moskovskogo Obshchestva Ispytateley Prirody, Zoologia i Botanika 1968–1969: 79–81 [In Russian].

31. Rasnitsyn AP (1972) On taxonomic analysis and some other taxometric methods. Zhurnal Obshchey Biologii 33(1): 60–76 [In Russian, with English summary].

32. Zhelochovtzev AN, Rasnitsyn AP (1972) On some Tertiary sawflies (Hymenoptera: Symphyta) from Colorado. Psyche 79(4): 315–327.

33. Rasnitsyn AP (1972) Praeaulacidae (Hymenoptera) from the Late Jurassic of Karatau. Paleontologicheskiy Zhurnal 1972(1): 70–87 [In Russian, with English translation in Paleontological Journal, 1972, 6(1): 62–77].

34. Rasnitsyn AP, Khovanov GM (1972) A method for more accurate estimation of the volume of local faunas. Paleontologicheskiy Zhurnal 1972(3): 162–167 [In Russian, with English translation in Paleontological Journal, 1972, 6(3): 422–426].

35. Rasnitsyn AP (1972) On the causes of morpho-functional progress. In: Zavadsky KM (Ed) Patterns of Progressive Evolution. Institute for the History of Science and Technology, Leningrad, 314–319 [In Russian].

36. Rasnitsyn AP (1973) *Praeaulacinus parvus *A. Rasnitsyn, nom. nov. Paleontologicheskiy Zhurnal 1973(1): 122 [In Russian, with English translation in Paleontological Journal, 1973, 7(1): 116].

37. Ponomarenko AG, Rasnitsyn AP (1974) New Mesozoic and Cenozoic Protomecoptera. Paleontologicheskiy Zhurnal 1974(4): 59–73 [In Russian, with English translation in Paleontological Journal, 1974, 8(4): 493–507].

38. Rasnitsyn AP (1975) On species and speciation. In: Vorontsov NN (Ed) Problems of Evolution. Volume 4. Nauka Press, Novosibirsk, 221–230 [total pages 238 pp.]. [In Russian].

39. Rasnitsyn AP (1975) Hymenoptera Apocrita of the Mesozoic. Trudy Paleontologicheskogo Instituta Akademii Nauk SSSR 147: 1–134 [In Russian].

40. Rasnitsyn AP (1975) Early evolution of higher Hymenoptera (Apocrita). Zoologicheskiy Zhurnal 54(6): 848–860 [In Russian, with English summary].

41. Rasnitsyn AP (1976) On the early evolution of insects and the origin of Pterygota. Zhurnal Obshchey Biologii 37(4): 543–555 [In Russian, with English summary].

42. Rasnitsyn AP (1976) Grylloblattidae are the living members of the order Protoblattodea. Doklady Akademii Nauk SSSR 228(2): 502–504 [In Russian, with English translation in Doklady Biological Sciences [Procedings of the Academy of Sciences of the USSR, Biological Sciences Section], 1976, 228: 273–275].

43. Rasnitsyn AP (1977) A new subfamily of scoliid wasps (Hymenoptera). Zoologicheskiy Zhurnal 56(4): 522–529 [In Russian, with English summary].

44. Rasnitsyn AP (1977) New Paleozoic and Mesozoic insects. Paleontologicheskiy Zhurnal 1977(1): 64–77 [In Russian, with English translation in Paleontological Journal, 1978, 11: 60–72].

45. Rasnitsyn AP (1977) New Hymenoptera from the Jurassic and Cretaceous of Asia. Paleontologicheskiy Zhurnal 1977(3): 98–108 [In Russian, with English translation in Paleontological Journal, 1978, 11: 349–357].

46. Rasnitsyn AP (1977) A new family of sawflies (Hymenoptera, Tenthredinoidea, Electrotomidae) from the Baltic amber. Zoologicheskiy Zhurnal 56(9): 1304–1308 [In Russian, with English summary].

47. Zherichin VV, Rasnitsyn AP (1978) “V.A. Krassilov. Evolution and biostratigraphy. Nauka Press, Moscow. 1977.” Zhurnal Obshchey Biologii 39(5): 798–800 [Book review; in Russian].

48. Rasnitsyn AP (1978) Introduction. In: Heinrich GH, Eastern Palearctic Ichneumoninae. Nauka Press, Leningrad, 3–5 [In Russian].

49. Kozlov MA, Rasnitsyn AP (1979) On the limits of the family Serphitidae (Hymenoptera, Proctotrupoidea). Entomologicheskoe Obozrenie 58(2): 402–416 [In Russian].

50. Rasnitsyn AP (1979) Morphological grounds of the new system of the insects. In: Sokolov VE (Ed) State and Prospects for Progress in Morphology. Nauka Press, Moscow, 382–383 [In Russian].

51. Rasnitsyn AP (1980) Origin and evolution of Hymenoptera. Trudy Paleontologicheskogo Instituta Akademii Nauk SSSR 174: 1–192 [In Russian].

52. Rohdendorf BB, Rasnitsyn AP, editors (1980) Historical development of the class Insecta. Trudy Paleontologicheskogo Instituta Akademii Nauk SSSR 175: 1–269, +8 pls. [In Russian]. [Note: The decision of how best to divide up the citations for Rasnitsyn’s individual contributions to this volume was based on those specifically identified and highlighted with his name in the table of contents on pp. 267–269.]

53. Rasnitsyn AP (1980) Class Scarabaeoda Laicharting, 1781. Trudy Paleontologicheskogo Instituta Akademii Nauk SSSR 175: 20–21 [In Russian]. [Rohdendorf BB, Rasnitsyn AP (Eds) Historical development of the class Insecta. Trudy Paleontologicheskogo Instituta Akademii Nauk SSSR, 1980, 175: 1–269].

54. Rasnitsyn AP (1980) Subclass Lepismatona [Latreille, 1804]. Trudy Paleontologicheskogo Instituta Akademii Nauk SSSR 175: 22–24 [In Russian]. [Rohdendorf BB, Rasnitsyn AP (Eds) Historical development of the class Insecta. Trudy Paleontologicheskogo Instituta Akademii Nauk SSSR, 1980, 175: 1–269].

55. Rasnitsyn AP (1980) Subclass Scarabaeona Laicharting, 1781. Trudy Paleontologicheskogo Instituta Akademii Nauk SSSR 175: 24–28 [In Russian]. [Rohdendorf BB, Rasnitsyn AP (Eds) Historical development of the class Insecta. Trudy Paleontologicheskogo Instituta Akademii Nauk SSSR, 1980, 175: 1–269].

56. Rasnitsyn AP (1980) Infraclass Scarabaeones Laicharting, 1781. Trudy Paleontologicheskogo Instituta Akademii Nauk SSSR 175: 29 [In Russian]. [Rohdendorf BB, Rasnitsyn AP (Eds) Historical development of the class Insecta. Trudy Paleontologicheskogo Instituta Akademii Nauk SSSR, 1980, 175: 1–269].

57. Rasnitsyn AP (1980) Cohort Paoliiformes Handlirsch, 1906. Trudy Paleontologicheskogo Instituta Akademii Nauk SSSR 175: 29–30 [In Russian]. [Rohdendorf BB, Rasnitsyn AP (Eds) Historical development of the class Insecta. Trudy Paleontologicheskogo Instituta Akademii Nauk SSSR, 1980, 175: 1–269].

58. Rasnitsyn AP (1980) Cohort Ephemeriformes Latreille, 1810. Trudy Paleontologicheskogo Instituta Akademii Nauk SSSR 175: 30–31 [In Russian]. [Rohdendorf BB, Rasnitsyn AP (Eds) Historical development of the class Insecta. Trudy Paleontologicheskogo Instituta Akademii Nauk SSSR, 1980, 175: 1–269].

59. Rasnitsyn AP (1980) Cohort Cimiciformes, Laicharting, 1781. Trudy Paleontologicheskogo Instituta Akademii Nauk SSSR 175: 36–38 [In Russian]. [Rohdendorf BB, Rasnitsyn AP (Eds) Historical development of the class Insecta. Trudy Paleontologicheskogo Instituta Akademii Nauk SSSR, 1980, 175: 1–269].

60. Rasnitsyn AP (1980) Superorder Caloneuridea Handlisch, 1906. Trudy Paleontologicheskogo Instituta Akademii Nauk SSSR 175: 38–41 [In Russian]. [Rohdendorf BB, Rasnitsyn AP (Eds) Historical development of the class Insecta. Trudy Paleontologicheskogo Instituta Akademii Nauk SSSR, 1980, 175: 1–269].

61. Rasnitsyn AP (1980) Superorder Hypoperlidea Martynov, 1928. Trudy Paleontologicheskogo Instituta Akademii Nauk SSSR 175: 41–43 [In Russian]. [Rohdendorf BB, Rasnitsyn AP (Eds) Historical development of the class Insecta. Trudy Paleontologicheskogo Instituta Akademii Nauk SSSR, 1980, 175: 1–269].

62. Rasnitsyn AP (1980) Superorder Dictyoneuridea Handlirsch, 1906. Trudy Paleontologicheskogo Instituta Akademii Nauk SSSR 175: 43–44 [In Russian]. [Rohdendorf BB, Rasnitsyn AP (Eds) Historical development of the class Insecta. Trudy Paleontologicheskogo Instituta Akademii Nauk SSSR, 1980, 175: 1–269].

63. Rasnitsyn AP (1980) Superorder Diaphanopteridea Handlirsch, 1906. Trudy Paleontologicheskogo Instituta Akademii Nauk SSSR 175: 49–52 [In Russian]. [Rohdendorf BB, Rasnitsyn AP (Eds) Historical development of the class Insecta. Trudy Paleontologicheskogo Instituta Akademii Nauk SSSR, 1980, 175: 1–269].

64. Rasnitsyn AP (1980) Cohort Scarabaeiformes Laicharting, 1781. Trudy Paleontologicheskogo Instituta Akademii Nauk SSSR 175: 72–74 [In Russian]. [Rohdendorf BB, Rasnitsyn AP (Eds) Historical development of the class Insecta. Trudy Paleontologicheskogo Instituta Akademii Nauk SSSR, 1980, 175: 1–269].

65. Rasnitsyn AP (1980) Superorder Palaeomanteida Handlirsch, 1906. Trudy Paleontologicheskogo Instituta Akademii Nauk SSSR 175: 74 [In Russian]. [Rohdendorf BB, Rasnitsyn AP (Eds) Historical development of the class Insecta. Trudy Paleontologicheskogo Instituta Akademii Nauk SSSR, 1980, 175: 1–269].

66. Rasnitsyn AP (1980) Superorder Papilionidea Laicharting, 1781. Trudy Paleontologicheskogo Instituta Akademii Nauk SSSR 175: 99–101 [In Russian]. [Rohdendorf BB, Rasnitsyn AP (Eds) Historical development of the class Insecta. Trudy Paleontologicheskogo Instituta Akademii Nauk SSSR, 1980, 175: 1–269].

67. Rasnitsyn AP (1980) Superorder Vespidea Laicharting, 1781. Trudy Paleontologicheskogo Instituta Akademii Nauk SSSR 175: 122–127 [In Russian]. [Rohdendorf BB, Rasnitsyn AP (Eds) Historical development of the class Insecta. Trudy Paleontologicheskogo Instituta Akademii Nauk SSSR, 1980, 175: 1–269].

68. Rasnitsyn AP (1980) Infraclass Gryllones Laicharting, 1781. Trudy Paleontologicheskogo Instituta Akademii Nauk SSSR 175: 134–135 [In Russian]. [Rohdendorf BB, Rasnitsyn AP (Eds) Historical development of the class Insecta. Trudy Paleontologicheskogo Instituta Akademii Nauk SSSR, 1980, 175: 1–269].

69. Rasnitsyn AP (1980) Superorder Blattidea Latreille, 1810. Trudy Paleontologicheskogo Instituta Akademii Nauk SSSR 175: 136–137 [In Russian]. [Rohdendorf BB, Rasnitsyn AP (Eds) Historical development of the class Insecta. Trudy Paleontologicheskogo Instituta Akademii Nauk SSSR, 1980, 175: 1–269].

70. Rasnitsyn AP (1980) Order Eoblattida Handlirsch, 1906. Trudy Paleontologicheskogo Instituta Akademii Nauk SSSR 175: 138 [In Russian]. [Rohdendorf BB, Rasnitsyn AP (Eds) Historical development of the class Insecta. Trudy Paleontologicheskogo Instituta Akademii Nauk SSSR, 1980, 175: 1–269].

71. Rasnitsyn AP (1980) Superorder Perlidea Latreille, 1802. Trudy Paleontologicheskogo Instituta Akademii Nauk SSSR 175: 148–150 [In Russian]. [Rohdendorf BB, Rasnitsyn AP (Eds) Historical development of the class Insecta. Trudy Paleontologicheskogo Instituta Akademii Nauk SSSR, 1980, 175: 1–269].

72. Rasnitsyn AP (1980) Order Grylloblattida Walker, 1914. Trudy Paleontologicheskogo Instituta Akademii Nauk SSSR 175: 150–154 [In Russian]. [Rohdendorf BB, Rasnitsyn AP (Eds) Historical development of the class Insecta. Trudy Paleontologicheskogo Instituta Akademii Nauk SSSR, 1980, 175: 1–269].

73. Rasnitsyn AP (1980) Order Perlida Latreille, 1802. Trudy Paleontologicheskogo Instituta Akademii Nauk SSSR 175: 154–160 [In Russian]. [Rohdendorf BB, Rasnitsyn AP (Eds) Historical development of the class Insecta. Trudy Paleontologicheskogo Instituta Akademii Nauk SSSR, 1980, 175: 1–269].

74. Rasnitsyn AP (1980) Superorder Geraridea Handlirsch, 1906. Trudy Paleontologicheskogo Instituta Akademii Nauk SSSR 175: 164–165 [In Russian]. [Rohdendorf BB, Rasnitsyn AP (Eds) Historical development of the class Insecta. Trudy Paleontologicheskogo Instituta Akademii Nauk SSSR, 1980, 175: 1–269].

75. Rasnitsyn AP (1980) Superorder Gryllidea Laicharting, 1781. Trudy Paleontologicheskogo Instituta Akademii Nauk SSSR 175: 165–166 [In Russian]. [Rohdendorf BB, Rasnitsyn AP (Eds) Historical development of the class Insecta. Trudy Paleontologicheskogo Instituta Akademii Nauk SSSR, 1980, 175: 1–269].

76. Rasnitsyn AP (1980) On the system of the family Aulacidae (Hymenoptera) in connection with a new finding in the Lower Cretaceous of Manlay. Trudy Sovmestnoy Sovetsko-Mongol’skoy Paleontologicheskoy Ekspeditsii 13: 65–67 [In Russian]. [Kalugina NS, editor (1980) The Early Cretaceous lake Manlay. Trudy Sovmestnoy Sovetsko-Mongol’skoy Paleontologicheskoy Ekspeditsii 13: 1–91].

77. Rasnitsyn AP (1980) “Keys for identification of insects of the European part of the USSR. Vol. III. Hymenoptera. Pt. 1. Nauka Press, Moscow. 1978.” Entomologicheskoe Obozrenie 59(2): 479–482 [Book review; in Russian].

78. Pulawski WJ, Rasnitsyn AP (1980) On the taxonomic position of *Hoplisus sepultus *Cockerell, 1906, from the Lower Oligocene of Colorado (Hymenoptera, Sphecidae). Polskie Pismo Entomologiczne 50(3): 393–396.

79. Zherichin VV, Rasnitsyn AP (1980) Biocoenotic regulation of the macroevolutionary process. In: Paaver KL, Sutt TY (Ed) Micro- and Macroevolution. Academy of Sciences of Estonian SSR, Tartu, 77–81 [total pages 236 pp.] [In Russian].

80. Alekseev VN, Rasnitsyn AP (1981) Late Cretaceous Megaspilidae (Hymenoptera) from amber of the Taimyr. Paleontologicheskiy Zhurnal 1981(4): 127–130 [In Russian, with English translation in Paleontological Journal, 1981, 15(4): 124–128].

81. Tichomirova AL, Rasnitsyn AP (1981) Recapitulations in ontogenesis of *Dolichovespa saxonica *(Hymenoptera, Vespidae). Zoologicheskiy Zhurnal 60(7): 1010–1023 [In Russian, with English summary].

82. Rasnitsyn AP, Siitan UV (1981) Subfam. Ichneumoninae. In: Kasparyan DR (Ed) Keys for Identification of Insects of the European Part of the USSR. Volume III. Hymenoptera. Part 3. Nauka Press, Moscow, 506–636 [In Russian].

83. Rasnitsyn AP (1981) “K.V. Krombein, P.D. Hurd, Jr., D.R. Smith, B.D. Burks. Catalog of Hymenoptera in America North of Mexico. Smiths. Inst. Press, Washington, D.C. 1979, vol. 1–2: 1–2209.” Entomologicheskoe Obozrenie 60(1): 231–233 [Book review; In Russian].

84. Rasnitsyn AP (1981) A modified paranotal theory of insect wing origin. Journal of Morphology 168(3): 331–338.

85. Rasnitsyn AP (1981) Gravenhorst’s and Berthoumieu’s types of Ichneumoninae Stenopneusticae preserved in Wrocław and Cracow, Poland (Hymenoptera, Ichneumonidae). Polskie Pismo Entomologiczne 51(1): 101–145.

86. Rasnitsyn AP (1982) Proposal to regulate the names of taxa above the family group. Z.N.(S.) 2381. Bulletin of Zoological Nomenclature 39(3): 200–207.

87. Rasnitsyn AP (1982) Triassic and Jurassic insects of the genus *Shurabia *(Grylloblattida, Geinitziidae). Paleontologicheskiy Zhurnal 1982(3): 78–87 [In Russian, with English translation in Paleontological Journal, 1982, 16(3): 77–86].

88. Krassilov VA, Rasnitsyn AP (1982) A unique finding: Pollen in the intestine of Early Cretaceous sawflies. Paleontologicheskiy Zhurnal 1982(4): 83–96 [In Russian, with English translation in Paleontological Journal, 1982, 16(4): 80–94].

89. Rasnitsyn AP (1983) Ichneumonoidea (Hymenoptera) from the Lower Cretaceous of Mongolia. Contributions of the American Entomological Institute 20: 259–265. [Gupta VK, editor (1983) Studies on the Hymenoptera: A collection of articles on Hymenoptera commemorating the 70th birthday of Henry K. Townes. Contributions of the American Entomological Institute 20: [i]+1–471].

90. Rasnitsyn AP (1983) Fossil Hymenoptera of the superfamily Pamphilioidea. Paleontologicheskiy Zhurnal 1983(2): 54–68 [In Russian, with English translation in Paleontological Journal, 1983, 17(2): 56–70].

91. Rasnitsyn AP (1983) New names for fossil insects. Paleontologicheskiy Zhurnal 1983(3): 103 [In Russian, with English translation in Paleontological Journal, 1983, 17(3): 99].

92. Rasnitsyn AP (1983) First finding of a moth in the Jurassic. Doklady Akademii Nauk SSSR 269(2): 467–471 [In Russian].

93. Rasnitsyn AP (1983) Phylogeny and taxonomy. In: Zykova LYu, Panov EN (Eds) Theoretical Problems of Modern Biology. Severtsov Institute of Evolutionary Morphology and Ecology of Animals RAS, Pushchino, 41–49 [In Russian].

94. Rasnitsyn AP (1983) Hymenopterous insects in the Jurassic of the eastern Siberia. Byulleten’ Moskovskogo Obshchestva Ispytateley Prirody, Otdel Biologicheskiy 58(5): 85–94 [In Russian].

95. Rasnitsyn AP (1984) Insects. In: Tatarinov LP, Shimansky VN (Eds) Handbook for the Taxonomy of Fossil Organisms. Nauka Press, Moscow, 81–84 [In Russian].

96. Rasnitsyn AP (1984) Living being as an adaptive compromise. In: Yanshin AL (Ed) Macroevolution (Materials of 1st All-Union Colloquium on Problems of Evolution). Nauka Press, Moscow, 233–234 [In Russian].

97. Rasnitsyn AP (1984) Type specimens of Ichneumoninae (Hymenoptera, Ichneumonidae) kept in Zoological Institute, Acad. Sci. USSR. I. Taxa described from the USSR territory. Entomologicheskoe Obozrenie 63(4): 790–801 [In Russian, with English translation in Entomological Review, 1984, 63(4): 115–126].

98. Melnikov OA, Rasnitsyn AP (1984) Zur Metamerie des Arthropoden-Kopfes: Das Acron. Beiträge zur Entomologie 34(1): 3–90.

99. Rasnitsyn AP, Kalugina NS, Ponomarenko AG (1984) Insects in the Mesozoic biocenoses of Siberia and Mongolia. In: Vasil’ev VP (Ed) 9th Congress of the All-Union Entomological Society. Kiev, October 1984. Pt. 2. Naukova Dumka, Kiev, 121–122.

100. Rasnitsyn AP (1985) *Eobraconus*, a substitute name for *Eobracon *Rasnitsyn (Hymenoptera, Braconidae). Psyche 92(1): 163.

101. Rasnitsyn AP, editor (1985) Jurassic insects of Siberia and Mongolia. Trudy Paleontologicheskogo Instituta Akademii Nauk SSSR 211: 1–192, +24 pls. [In Russian].

102. Rasnitsyn AP, editor (1985) Jurassic continental biocoenoses of southern Siberia and adjacent territories*. *Trudy Paleontologicheskogo Instituta Akademii Nauk SSSR 213: 1–198, +8 pls. [In Russian].

103. Rasnitsyn AP (1986) Type specimens of Ichneumoninae (Hymenoptera, Ichneumonidae) kept in Zoological Institute, Acad. Sci. USSR. II. Species described by N.R. Kokujev from China. Entomologicheskoe Obozrenie 65(1): 142–152 [In Russian, with English translation in Entomological Review, 1986, 65(1): 142–152].

104. Rasnitsyn AP (1986) New species of the family Mesoserphidae (Hymenoptera) from the Upper Jurassic of Karatau. Vestnik Zoologii 1986(2): 19–25 [In Russian].

105. Rasnitsyn AP, editor (1986) Insects in the Early Cretaceous ecosystems of the West Mongolia. Trudy Sovmestnoy Sovetsko-Mongolskoy Paleontologicheskoy Ekspeditsii 28: 1–213, +24 pls. [In Russian].

106. Rasnitsyn AP (1986) Vespida (= Hymenoptera). Trudy Sovmestnoy Sovetsko-Mongol’skoy Paleontologicheskoy Ekspeditsii 28: 154–164 [In Russian]. [Rasnitsyn AP, editor (1986) Insects in the Early Cretaceous ecosystems of the West Mongolia. Trudy Sovmestnoy Sovetsko-Mongol’skoy Paleontologicheskoy Ekspeditsii 28: 1–213, +24 pls.].

107. Rasnitsyn AP (1986) Review of the fossil Tiphiidae, with description of a new species (Hymenoptera). Psyche 93(1–2): 91–101.

108. Rasnitsyn AP (1986) Inadaptation and euadaptation. Paleontologicheskiy Zhurnal 1986(1): 3–7 [In Russian, with English translation in Paleontological Journal, 1987, 20(1): 1–4].

109. Rasnitsyn AP (1986) Parataxon and paranomenclature. Paleontologicheskiy Zhurnal 1986(3): 11–21 [In Russian, with English translation in Paleontological Journal, 1987, 20(3): 25–33].

110. Tatarinov LP, Rasnitsyn AP, editors (1987) Evolution and Biocoenotic Crises. Nauka Press, Moscow, 159 pp. [In Russian].

111. Rasnitsyn AP (1987) Tempo of evolution and evolutionary theory (hypothesis of the adaptive compromise). In: Tatarinov LP, Rasnitsyn AP (Eds) Evolution and Biocenotic Crises. Nauka Press, Moscow, 46–64 [total pages 159 pp.] [In Russian].

112. Menke AS, Rasnitsyn AP (1987) Affinities of the fossil wasp, *Hoplisidea kohliana *Cockerell (Hymenoptera: Sphecidae: Sphecinae). Psyche 94(1–2): 35–38.

113. Rasnitsyn AP (1987) The importance of [not] being a cladist. Sphecos 14: 23–25.

114. Rasnitsyn AP (1988) Sepulcidae and origin of Cephidae (Hymenoptera: Cephoidea). Transactions of the All-Union Entomological Society 70: 480–497 [In Russian]. [Tobias VI, editor (1988) Taxonomy of Insects and Mites. Nauka Press, Moscow].

115. Rasnitsyn AP (1988) Phylogenetics. In: Menner VV, Makridin VP (Eds) Modern Paleontology [Volume I]. Nedra Press, Moscow, 480–497 [In Russian].

116. Rasnitsyn AP, Dlussky GM (1988) Principles and methods of phylogeny reconstruction. In: Ponomarenko AG (Ed) The Cretaceous Biocoenotic Crisis and the Evolution of Insects. Nauka Press, Moscow, 5–15 [total pages 227+[5] pp., +4pls.] [In Russian].

117. Rasnitsyn AP (1988) Problem of the global crisis of non-marine biocoenoses in the mid-Cretaceous. In: Ponomarenko AG (Ed) The Cretaceous Biocoenotic Crisis and the Evolution of Insects. Nauka Press, Moscow, 191–207 [total pages 227+[5] pp., +4pls.] [In Russian]

118. Rasnitsyn AP, Kovalev OV (1988) The oldest gall-wasps from the Early Cretaceous of Transbaikalia (Hymenoptera, Cynipoidea, Archaeocynipidae fam. nov.). Vestnik Zoologii 1988(1): 18–21 [In Russian].

119. Rasnitsyn AP (1988) On the Red Queen, progression of reproduction and group selection. In: Krassilov VA (Ed) Evolutionary Studies: Vavilov’s Themes. Institute of Biology and Soil Sciences FEB RAS, Vladivostok, 47–53 [In Russian].

120. Rasnitsyn AP (1988) An outline of evolution of the hymenopterous insects (order Vespida). Oriental Insects 22: 115–145.

121. Rasnitsyn AP (1988) Paleontological succession of hymenopterans. In: Proceedings of the XVIII International Congress of Entomology, Vancouver 1988: 9.

122. Tichomirova AL, Rasnitsyn AP (1988) Recapitulations in holometabolan morphogenesis. In: Proceedings of the XVIII International Congress of Entomology, Vancouver 1988: 73.

123. Rasnitsyn AP, Sharkey M[J] (1988) New Eoichneumonidae from Early Cretaceous of Siberia and Mongolia (Hymenoptera: Ichneumonoidea). In: Gupta VK (Ed) Advances in Parasitic Hymenoptera Research. Brill, Leiden, 169–197 [total pages [iv]+546 pp.].

124. Babak EV, Goncharova IA, Rasnitsyn AP (1988) Dynamics of speciation and extinction of the Neogene bivalve molluscs in eastern Paratethys. In: Severtzov AS (Ed) Problems of Macroevolution. Nauka Press, Moscow, 4–5 [In Russian].

125. Goncharova IA, Rasnitsyn AP (1988) Evolution of bivalve molluscs in the Miocene of Paratethys as reflected in the Layellian curves. In: Severtzov AS (Ed) Problems of Macroevolution. Nauka Press, Moscow, 9 [In Russian].

126. Rasnitsyn AP, editor (1988) Insects of the Moscow Region: Problems of Land Surveying and Conservation. Nauka Press, Moscow, 158 pp. [In Russian].

127. Rasnitsyn AP (1989) Dynamics of insect families and a hypothesis of the Cretaceous biocoenotic crisis. In: Sokolov BS (Ed) Sediment Cover of the Earth in Space and Time: Stratigraphy and Paleontology. Nauka Press, Moscow, 35–40 [In Russian, with English summary].

128. Rasnitsyn AP (1989) Haldane’s nightmare is a fiction. A remark to B.M. Mednikov. Zhurnal Obshchey Biologii 50(1): 136–137 [In Russian].

129. Rasnitsyn AP (1989) Phytospreading from the selectionist’s viewpoint. Zhurnal Obshchey Biologii 50(5): 581–583 [In Russian, with English summary].

130. Rasnitsyn AP (1989) Remark to remark, or once more about the transition load. Zhurnal Obshchey Biologii 50(5): 719 [In Russian].

131. Rasnitsyn AP, Matveev DG (1989) The first Palearctic representative of the genus *Ampulicomorpha *Ashmead (Hymenoptera, Embolemidae). Entomologicheskoe Obozrenie 68(3): 657–661 [In Russian, with English translation in Entomological Review, 1990, 69(3): 133–137].

132. Rasnitsyn AP (1989) Vespida vs. Hymenoptera. Sphecos 18: 8.

133. Rasnitsyn AP (1990) History of paleoentomology and history of insects. Priroda 1990(6): 66–80 [In Russian].

134. Rasnitsyn AP, Kozlov MV (1990) A new group of fossil insects: Scorpion-fly with cicada and moth adaptations. Doklady Akademii Nauk SSSR 310: 973–976 [In Russian, with English translation in Transactions (Doklady) of the USSR Academy of Sciences: Earth Science Sections 1991, 310: 233–236].

135. Rasnitsyn AP, editor (1990) Late Mesozoic insects of eastern Transbaikalia. Trudy Paleontologicheskogo Instituta Akademii Nauk SSSR 239: 1–221, +16 pls. [In Russian].

136. Rasnitsyn AP (1990) Vespida. Hymenoptera. Trudy Paleontologicheskogo Instituta Akademii Nauk SSSR239: 177–205 [In Russian]. [Rasnitsyn AP, editor (1990) Late Mesozoic insects of eastern Transbaikalia. Trudy Paleontologicheskogo Instituta Akademii Nauk SSSR 239: 1–221, +16 pls.].

137. Rasnitsyn AP (1990) Table 1 [Known Cretaceous Hymenoptera]. In: Darling DC, Sharkey MJ (Authors) Order Hymenoptera. Bulletin of the American Museum of Natural History 195: 124–129. [Grimaldi DA, editor (1990), Insects from the Santana Formation, Lower Cretaceous, of Brazil. Bulletin of the American Museum of Natural History, 195, 1–191].

138. Rasnitsyn AP (1990) Hymenopterous insects and stratigraphy of the Cretaceous. In: Krassilov VA (Ed) Continental Cretaceous of the USSR. Far Eastern Branch of the USSR Academy of Sciences, Vladivostok, 15–18 [In Russian].

139. Rasnitsyn AP (1990) Rank problem in taxonomy. In: Menner VV (Ed) Taxonomy and Phylogeny of Invertebrata. Nauka Press, Moscow, 5–10 [In Russian, with English summary].

140. Rasnitsyn AP (1990) New representatives of the hymenopterous family Praeaulacidae from Early Cretaceous Buryatia and Mongolia. Vestnik Zoologii 1990(6): 27–31 [In Russian].

141. Carpenter JM, Rasnitsyn AP (1990) Mesozoic Vespidae. Psyche 97(1–2): 1–20.

142. Rasnitsyn AP, Kulicka R (1990) Hymenopteran insects in Baltic amber with respect to the overall history of the order. Prace Muzeum Ziemi 41: 53–64.

143. Rasnitsyn AP (1991) New hymenopterans of the genus *Baissa *(Gasteruptiidae s.l.) from the Early Cretaceous in Buryatia and Mongolia. Vestnik Zoologii 1991(1): 78–79 [In Russian, with English summary].

144. Emelyanov AF, Rasnitsyn AP (1991) Systematics, taxonomy, cladistics. Priroda 1991(7): 26–37 [In Russian].

145. Rasnitsyn AP (1991) Patronage of pure science. Priroda 1991(8): 88–89 [In Russian].

146. Rasnitsyn AP (1991) Early Cretaceous members of evaniomorphous hymenopteran families Stigmaphronidae, Cretevaniidae, and subfamily Kotujellitinae (Gasteruptiidae). Paleontologicheskiy Zhurnal 1991(4): 128–132 [In Russian, with English translation in Paleontological Journal, 1991, 25(4): 172–179].

147. Rasnitsyn AP, Michener CD (1991) Miocene fossil bumble bee from the Soviet Far East with comments on the chronology and distribution of fossil bees (Hymenoptera: Apidae). Annals of the Entomological Society of America 84(6): 583–589.

148. Rasnitsyn AP (1991) Typified names of higher taxa once again. Sphecos 22: 5–7.

149. Rasnitsyn AP (1991) Nomenclature for the XXI century? Sphecos 22: 7–8.

150. [Rasnitsyn AP, editor] Tichomirova AL (1991) Ontogeny Transformations as a Mechanism of Insect Evolution. Nauka Press, Moscow, 168 pp. [In Russian].

151. Sukacheva ID, Rasnitsyn AP (1992) First members of the family Boreidae (Insecta, Panorpida) from the Upper Jurassic of Mongolia and the Lower Cretaceous of Transbaikalia. Paleontologicheskiy Zhurnal 1992(1): 126–129 [In Russian, with English translation in Paleontological Journal, 1992, 26: 168–172].

152. Rasnitsyn AP (1992) Principles of phylogenetics and taxonomy. Zhurnal Obshchey Biologii 53(2): 172–185 [In Russian].

153. Rasnitsyn AP (1992) Principles of nomenclature and the nature of taxon. Zhurnal Obshchey Biologii 53(3): 307–313 [In Russian].

154. Rasnitsyn AP (1992) Comment on the article *Problems in the Nomenclature of Higher Taxonomic Categories *by Ya.I. Starobogatov (see BZN 48: 6–18). Bulletin of Zoological Nomenclature 49(1): 62.

155. Kasparyan DR, Rasnitsyn AP (1992) On the systematic position of the Oligocene *Lithoserix williamsi *Brown (Hymenoptera: Ichneumonidae) from Colorado (USA). Paleontologicheskiy Zhurnal 1992(3): 113–114 [In Russian, with English translation in Paleontological Journal, 1992, 26(3): 134–135].

156. Melnikov OA, Eskov KY, Rasnitsyn AP (1992) On the promorphology of chelicerates. Izvestiya Akademii Nauk, Seriya Biologicheskaya 1992(3): 405–415 [In Russian, with English summary].

157. Rasnitsyn AP, Sinitshenkova ND (1992) Insects in deposits of the Vorkuta series. In: Abstracts of the All-Russian Conference on Paleontology and Stratigraphy of Non-Marine Deposits of the Permian and Triassic of North Eurasia. Paleontological Institute, Russian Academy of Sciences, Moscow, 15–16 [In Russian].

158. Rasnitsyn AP (1992) *Strashila incredibilis*, a new enigmatic mecopteroid insect with possible siphonateran affinities from the Upper Jurassic of Siberia. Psyche 99(4):319–329.

159. Rasnitsyn AP (1993) George Soros Grant competition in biodiversity: Preliminary results. Zhurnal Obshchey Biologii 54(3): 382–383 [In Russian].

160. Rasnitsyn AP (1993) George Soros Grant competition in biodiversity: Preliminary results. Zoologicheskiy Zhurnal 72(6): 152–153 [In Russian].

161. Rasnitsyn AP (1993) George Soros Grant competition in biodiversity (preliminary results). Paleontologicheskiy Zhurnal 1993(1): 141–142 [In Russian].

162. Gromov VP, Dmitriev VYu, Zherikhin VV, Lebedev EL, Ponomarenko AG, Rasnitsyn AP, Sukacheva ID (1993) Cretaceous insect faunas from Ul’ya River basin (West Okhotsk region). Trudy Paleontologicheskogo Instituta RossiyskoyAkademii Nauk 252: 5–60 [In Russian]. [Ponomarenko AG, editor (1993) Mesozoic insects from Uljya River Basin (West Okhotsk Region). Mesozoic Insects and Ostracods from Asia, Trudy Paleontologicheskogo Instituta RossiyskoyAkademii Nauk 252: 1–159, +16 pls.].

163. Rasnitsyn AP (1993) New taxa of Sepulcidae (Vespida). Trudy Paleontologicheskogo Instituta RossiyskoyAkademii Nauk 252: 80–99 [In Russian]. [Ponomarenko AG, editor (1993) Mesozoic Insects and Ostracods from Asia. Trudy Paleontologicheskogo Instituta RossiyskoyAkademii Nauk 252: 1–159, +16 pls.]

164. Rasnitsyn AP (1993) Archaeoscoliinae, an extinct subfamily of scoliid wasps (Insecta: Vespida = Hymenoptera: Scoliidae). Journal of Hymenoptera Research 2: 85–96.

165. Brothers DJ, Rasnitsyn AP (1993) Mesozoic aculeate Hymenoptera from Orapa, Botswana. In: Proceedings of the 9th Entomological Congress, Entomological Society of Southern Africa, Johannesburg 28 June–1 July 1993. Entomological Society of Southern Africa, Pretoria, 132.

166. Dmitriev VY, Ponomarenko AG, Rasnitsyn AP (1994) Dynamics of the taxonomic diversity of nonmarine biota. In: Rozanov AYu, Semikhatov MA (Eds) Ecosystem Restructures and the Evolution of Biosphere. Nedra Press, Moscow, 167–174 [In Russian].

167. Dmitriev VY, Ponomarenko AG, Rasnitsyn AP (1994) Organic diversity in geological past. State of the problem. In: Sokolov VE, Reshetnikov YuS (Eds) Biodiversity: Degree of Taxonomic Knowledge. Nauka Press, Moscow, 12–19 [In Russian].

168. Rasnitsyn AP (1994) New Late Jurassic Mesoserphidae (Vespida, Proctotrupoidea). Paleontologicheskiy Zhurnal 1994(2): 115–119 [In Russian, with English translation in Paleontological Journal, 1994, 28: 141–147].

169. Rasnitsyn AP (1995) Tertiary sawflies of the tribe Xyelini (Insecta: Vespida = Hymenoptera: Xyelidae) and their relationship to the Mesozoic and modern faunas. Contributions in Science 450: 1–14.

170. Rasnitsyn AP, Krassilov VA (1995) First finding of pollen in intestines of Permian insects. In: Ecosystem Evolution: International Symposium, Moscow, 26–30 September 1995: Abstracts. Paleontological Institute, Russian Academy of Sciences, Moscow, 76, 101–102 [Russian and English versions of text].

171. Rasnitsyn AP (1995) Lower Cretaceous hymenopterans from the English Weald and Purbeck. In: International Society of Hymenopterists Third Annual Conference: Abstracts of Papers and Posters, August 12–17, 1995, University of California, Davis, California. University of California Davis, Davis, 24 [total pages 34 pp.]

172. Dmitriev VY, Ponomarenko AG, Rasnitsyn AP (1995) Dynamics of the taxonomic diversity of nonmarine aquatic biota. Paleontologicheskiy Zhurnal 1995(4): 3–9 [In Russian, with English translation in Paleontological Journal, 1996, 30(3): 255–259].

173. Rasnitsyn AP (1996) Conceptual issues in phylogeny, taxonomy, and nomenclature. Contributions to Zoology 66(1): 3–41.

174. Rasnitsyn AP (1996) New Early Cretaceous Embolemidae (Vespida = Hymenoptera: Chrysidoidea). Memoirs of the Entomological Society of Washington 17: 183–187. [Norden BB, Menke AS, editors (1996) Contributions on Hymenoptera and associated insects dedicated to Karl V. Krombein. Memoirs of the Entomological Society of Washington 17: 1–216].

175. Rasnitsyn AP (1996) Phylogenetic problems in the Insecta. In: International Symposium on the Relationships of Major Arthropod Groups: Programme and Abstracts, 17–19 April, 1996. The Natural History Museum, London, 16.

176. Rasnitsyn AP (1996) Burmese amber at the Natural History Museum. Wrostek (Inclusion) 23: 19–20.

177. Rasnitsyn AP, Krassilov VA (1996) First find of pollen grains in the gut of Permian insects. Paleontologicheskiy Zhurnal 1996(3): 119–124 [In Russian, with English translation in Paleontological Journal, 1996, 30(4): 484–490].

178. Rasnitsyn AP, Krassilov VA (1996) Pollen in the gut contents of fossil insects as evidence of coevolution. Paleontological Journal 30(6): 716–722.

179. Krassilov VA, Rasnitsyn AP (1997) Pollen in the guts of Permian insects: First evidence of pollinivory and its evolutionary significance. Lethaia 29(4): 369–372.

180. Rasnitsyn AP, Novokshonov VG (1997) On the morphology of *Uralia maculata *(Insecta: Diaphanopterida) from the Early Permian (Kungurian) of Ural (Russia). Entomologica Scandinavica 28(1): 27–38.

181. Rasnitsyn AP (1997) *Xyela* (*Pinicolites*) *lata *Smith (Vespida: Xyelidae), a living fossil sawfly from western North America. Pan-Pacific Entomologist 73(4): 231–235.

182. Dobruskina IA, Ponomarenko AG, Rasnitsyn AP (1997) Fossil insect findings in Israel. Paleontologicheskiy Zhurnal 1997(5): 91–95 [In Russian, with English translation in Paleontological Journal, 1997, 31(5): 528–533].

183. Krassilov VA, Zherikhin VV, Rasnitsyn AP (1997) Pollen in guts of fossil insects as evidence for coevolution. Doklady Akademii Nauk 354(1): 135–138 [In Russian, with English translation in Doklady Biological Sciences, 1997, 354: 239–241].

184. Krassilov VA, Zherikhin VV, Rasnitsyn AP (1997) *Classopollis* in the guts of Jurassic insects. Palaeontology 40(4): 1095–1101.

185. Rasnitsyn AP (1998) Problem of the basal dichotomy of the winged insects. In: Fortey RA, Thomas RH (Eds) Arthropod Relationships. Chapman and Hall, London, 237–248 [total pages xii+383 pp.].

186. Rasnitsyn AP, Jarzembowski EA, Ross AJ (1998) Wasps (Insecta: Vespida = Hymenoptera) from the Purbeck and Wealden (Lower Cretaceous) of southern England and their biostratigraphical and palaeoenvironmental significance. Cretaceous Research 19(3–4): 329–391.

187. Rasnitsyn AP (1998) On the taxonomic position of the insect order Zorotypida = Zoraptera. Zoologischer Anzeiger 237(2–3): 185–194.

188. Martínez-Delclòs X, Peñalver-Mollá E, Rasnitsyn A[P] (1998) Los Hymenoptera del ámbar del Cretácico inferior de Álava (País Vasco, España). In: World Congress on Amber Inclusions, 20–23 October 1998, Vitoria-Gasteiz, Álava-Araba Basque Country. Álava Museo de Ciencias Naturales de Álava, Vitoria-Gasteiz, 119.

189. Martínez-Delclòs X, Peñalver-Mollá E, Rasnitsyn A[P] (1998) Serphitidae (Insecta: Hymenoptera) from the Lower Cretaceous amber of Álava (Spain). In: World Congress on Amber Inclusions, 20–23 October 1998, Vitoria-Gasteiz, Álava-Araba Basque Country. Álava Museo de Ciencias Naturales de Álava, Vitoria-Gasteiz, 161.

190. Krassilov VA, Rasnitsyn AP, Afonin SA (1999) Pollen morphotypes from the intestine of a Permian booklouse. Review of Palaeobotany and Palynology 106(1–2): 89–96.

191. Rasnitsyn AP, Pulawski WJ, Martínez-Delclòs X (1999) Cretaceous digger wasps of the new genus *Bestiola* Pulawski and Rasnitsyn (Hymenoptera: Sphecidae: Angarosphecinae). Journal Hymenoptera Research 8(1): 23–34.

192. Rasnitsyn AP (1999) Insect taxonomy. In: Grodnitsky DL (Author) Form and Function of Insect Wings: The Evolution of Biological Structures. John Hopkins University Press, Baltimore, 1–11 [total pages xiv+[i]+261 pp.].

193. Rasnitsyn AP (1999) “P.A. Lehr (ed.). Keys to the insects of the Far East of Russia. Vol. IV. Neuropteroidea, Mecoptera, Hymenoptera. A.S. Lelej (ed.). Pt. 1. St-Petersburg, Nauka. 1995. 607 p. A.N. Kupyanskaya (ed.) Pt. 2. Vladivostok: Dal’nauka. 1995. 599 p. A.S. Lelej (ed.) Pt. 3. Vladivostok: Dal’nauka. 1998. 708 p.” Zoologicheskiy Zhurnal 78(8): 1021–1022 [Book review; In Russian].

194. Ronquist F, Rasnitsyn AP, Roy A, Eriksson K, Lindgren M (1999) Phylogeny of the Hymenoptera: A cladistic reanalysis of Rasnitsyn’s (1988) data. Zoologica Scripta28(1–2): 13–50.

195. Quicke DLJ, Basibuyuk HH, Fitton MG, Rasnitsyn AP (1999) Morphological, palaeontological and molecular aspects of ichneumonoid phylogeny (Hymenoptera, Insecta). Zoologica Scripta28(1–2): 175–202.

196. Basibuyuk HH, Rasnitsyn AP, Achterberg K van, Fitton MG, Quicke DLJ (1999) A new, putatively primitive Cretaceous fossil braconid subfamily from New Jersey amber (Hymenoptera, Braconidae). Zoologica Scripta28(1–2): 211–214.

197. Rasnitsyn AP, Martínez-Delclòs X (1999) New Cretaceous Scoliidae (Vespida = Hymenoptera) from the Lower Cretaceous of Spain and Brazil. Cretaceous Research 20(6): 767–772.

198. Martínez-Delclòs X, Peñalver-Mollá E, Rasnitsyn A[P] (1999) Hymenopteran insects from the Lower Cretaceous amber of Alava (Spain). In: Abstracts of the VII International Symposium on Mesozoic Terrestrial Ecosystems, Buenos Aires, 1999. Asociación Paleontológica Argentina, Buenos Aires, 42.

199. Dlussky GM, Rasnitsyn AP (1999) Two new species of aculeate hymenopterans (Vespida = Hymenoptera) from the middle Eocene of the United States. Paleontologicheskiy Zhurnal 1999(5): 72–75 [In Russian, with English translation in Paleontological Journal, 1999, 33(5): 546–549].

200. Krassilov VA, Rasnitsyn AP (1999) Plant remains from the guts of fossil insects: Evolutionary and paleoecological inferences. In: Vršanský P (Ed) Proceedings of the First Palaeoentomological Conference Moscow 1998. AMBA Projects International, Bratislava, 65–72 [total pages 199 pp.].

201. Rasnitsyn AP (1999 [2000]).*Cratephialtites *gen. nov. (Vespida = Hymenoptera: Ephialtitidae), a new genus for *Karataus koiurus *Sharkey, 1990, from the Lower Cretaceous of Brazil. Russian Entomological Journal 8(2): 135–136.

202. Rasnitsyn AP (1999 [2000]). Taxonomy and morphology of *Dasyleptus *Brongniart, 1885, with description of a new species (Insecta: Machilida: Dasyleptidae). Russian Entomological Journal8(3): 145–154.

203. Rasnitsyn AP, Zherikhin VV (1999 [2000]). First fossil chewing louse from the Lower Cretaceous of Baissa, Transbaikalia (Insecta, Pediculida = Phthiriaptera, Saurodectidae fam. n.). Russian Entomological Journal 8(4): 253–255.

204. Basibuyuk HH, Quicke DLJ, Rasnitsyn AP (2000) A new genus of the Orussidae (Insecta: Hymenoptera) from Late Cretaceous New Jersey amber. In: Grimaldi D (Ed) Studies on Fossils in Amber, with Particular Reference to the Cretaceous of New Jersey. Backhuys Publishers, Leiden, 305–311 [total pages vi+498 pp.].

205. Basibuyuk HH, Fitton MG, Rasnitsyn AP, Quicke DLJ (2000) Two new genera of the Evaniidae (Insecta: Hymenoptera) from Late Cretaceous New Jersey amber. In: Grimaldi D (Ed) Studies on Fossils in Amber, with Particular Reference to the Cretaceous of New Jersey. Backhuys Publishers, Leiden, 313–325 [total pages vi+498 pp.].

206. Rasnitsyn AP (2000) An extremely primitive aculeate wasp in the Cretaceous amber from New Jersey (Vespida: ?Sierolomorphidae). In: Grimaldi D (Ed) Studies on Fossils in Amber, with Particular Reference to the Cretaceous of New Jersey. Backhuys Publishers, Leiden, 327–332 [total pages vi+498 pp.].

207. Rasnitsyn AP, Krassilov VA (2000) The first documented occurrence of phyllophagy in pre-Cretaceous insects: Leaf tissues in the gut of Upper Jurassic insects from southern Kazakhstan. Paleontologicheskiy Zhurnal 2000(3): 73–81 [In Russian, with English translation in Paleontological Journal, 2000, 34(3): 301–309].

208. Pulawski WJ, Rasnitsyn AP, Brothers DJ, Archibald SB (2000) New genera of Angarosphecinae: *Cretosphecium *from Early Cretaceous of Mongolia and *Eosphecium *from Early Eocene of Canada (Hymenoptera: Sphecidae). Journal of Hymenoptera Research 9(1): 34–40.

209. Pulawski WJ, Rasnitsyn AP (2000) *Cretobestiola*, a replacement name for *Bestiola *Pulawski and Rasnitsyn, 1999 (Hymenoptera: Sphecidae). Journal of Hymenoptera Research 9(1): 209.

210. Ansorge J, Rasnitsyn AP (2000) Identity of *Prosepididontus calopteryx *Handlirsch 1920 (Insecta: Grylloblattida: Geinitziidae). Acta Geológica Hispánica 35(1–2): 19–23. [Martínez-Delclòs X, Nel A (Eds), Studies on Mesozoic and Tertiary Insects. Systematics, Phylogeny and Taphonomy. Acta Geológica Hispánica, 2000, 35(1–2): 1–193].

211. Pulawski WJ, Rasnitsyn AP (2000) *Cretobestiola*, a replacement name for *Bestiola *Pulawski and Rasnitsyn, 1999 (Hymenoptera: Sphecidae). Acta Geológica Hispánica 35(1–2): 53. [Martínez-Delclòs X, Nel A (Eds), Studies on Mesozoic and Tertiary Insects. Systematics, Phylogeny and Taphonomy. Acta Geológica Hispánica, 2000, 35(1–2): 1–193].

212. Rasnitsyn AP (2000) New genus and two new species of the Lower Cretaceous digger wasps from Spain (Hymenoptera: Sphecidae, Angarosphecidae). Acta Geológica Hispánica 35(1–2): 55–58. [Martínez-Delclòs X, Nel A (Eds), Studies on Mesozoic and Tertiary Insects. Systematics, Phylogeny and Taphonomy. Acta Geológica Hispánica, 2000, 35(1–2): 1–193].

213. Rasnitsyn AP, Ansorge J (2000) Two new Lower Cretaceous hymenopterous insects (Insecta: Hymenoptera) from Sierra del Montsec, Spain. Acta Geológica Hispánica 35(1–2): 59–64. [Martínez-Delclòs X, Nel A (Eds), Studies on Mesozoic and Tertiary Insects. Systematics, Phylogeny and Taphonomy. Acta Geológica Hispánica, 2000, 35(1–2): 1–193].

214. Rasnitsyn AP, Martínez-Delclòs X (2000) Wasps (Insecta: Vespida = Hymenoptera) from the Early Cretaceous of Spain. Acta Geológica Hispánica 35(1–2): 65–95. [Martínez-Delclòs X, Nel A (Eds), Studies on Mesozoic and Tertiary Insects. Systematics, Phylogeny and Taphonomy. Acta Geológica Hispánica, 2000, 35(1–2): 1–193].

215. Rasnitsyn AP, Ross AJ (2000) A preliminary list of arthropod families present in the Burmese amber collection at The Natural History Museum, London. Bulletin of the Natural History Museum, Geology Series 56(1): 21–24. [Ross AJ (Ed) The History, Geology, Age and Fauna (Mainly Insects) of Burmese Amber, Myanmar. Bulletin of the Natural History Museum, Geology Series, 56(1): 1–83].

216. Basibuyuk HH, Rasnitsyn AP, Fitton MG, Quicke DLJ (2000) An archaic new genus of Evaniidae (Insecta: Hymenoptera) and implications for the biology of ancestral evanioids. Bulletin of the Natural History Museum, Geology Series 56(1): 53–58. [Ross AJ (Ed) The History, Geology, Age and Fauna (Mainly Insects) of Burmese Amber, Myanmar. Bulletin of the Natural History Museum, Geology Series, 56(1): 1–83].

217. Brothers DJ, Rasnitsyn AP (2000) Evolution of the enigmatic Plumariidae (Insecta: Hymenoptera: Chrysidoidea). In: Multidisciplinary Approaches to Systematics [Second Conference of the Southern African Society for Systematic Biology, 10–14 July 2000]. Southern African Society for Systematic Biology, Mtunzini, 24.

218. Basibuyuk HH, Quicke DLJ, Rasnitsyn AP, Fitton MG (2000) Morphology and sensilla of the orbicula, a sclerite between the tarsal claws, in the Hymenoptera. Annals of the Entomological Society of America93(3): 625–636.

219. Basibuyuk HH, Rasnitsyn AP, Fitton MG, Quicke DLJ (2000) Hymenopteran orbicular sensilla. In: Austin AD, Dowton M (Eds) Hymenoptera: Evolution, Biodiversity and Biological Control. CSIRO Publishing, Melbourne, 192–197 [total pages xi+468 pp.].

220. Novokshonov VG, Rasnitsyn AP (2000) A new enigmatic group of insects (Psocidea, Tshekarcephalidae) from Tshekarda (Lower Permian of the Middle Urals). Paleontological Journal34(Supplement 3): S284–S287.

221. Rasnitsyn AP, Jarzembowski EA (2000) A replacement name for the parasitoid wasp *Arossia* Rasnitsyn et Jarzembowski non Newman. Cretaceous Research 21(4): 587.

222. Rasnitsyn AP (2000) Testing cladograms by fossil record: The ghost range test. Contributions to Zoology69(4): 251–258.

223. Rasnitsyn AP, Ansorge J (2000) New Early Cretaceous hymenopterous insects (Insecta: Hymenoptera) from Sierra del Montsec (Spain). Paläontologische Zeitschrift74(3): 335–341.

224. Rasnitsyn AP (2001) [unnamed in editorial] Comments on Article 74.7.3 of the Code (requirement for an express statement of the taxonomic purpose of a lectotype designation), including a proposal that it should be revoked. Bulletin of Zoological Nomenclature 58(2): 136.

225. Rasnitsyn AP (2001) On the skimming hypothesis of insect flight origin. In: Krzemińska E, Krzemiński W (Eds) Fossil Insects [Second International Congress on Palaeoentomology, 5–9 September, 2001, Kraków, Poland: Abstracts Volume]. Polish Academy of Science, Kraków, 56–57 [total pages 94 pp.].

226. Rasnitsyn AP, Basibuyuk HH, Quicke DLJ (2001) A putative extinct stem chalcidoid family from the lowermost Cretaceous or uppermost Jurassic in Mongolia (Hymenoptera, Chalcidoidea). In: Olckers T, Brothers DJ (Eds) Proceedings of the 13th Entomological Congress [Organised by the Entomological Society of Southern Africa, 2–5 July 2001, Pietermaritzburg]. Entomological Society of Southern Africa, Hatfield, 55 [total pages 114 pp.].

227. Vršanský P, Quicke DLJ, Rasnitsyn AP, Basibuyuk HH, Ross AJ, Fitton MG, Vidlička L (2001) The oldest fossilinsect sensilla. AMBA/B/21.01.3/ABS/D: 1–8.

228. Rasnitsyn AP (2001) [unnamed in editorial] Comments on the proposed revocation of Article 74.7.3 of the Code. Bulletin of Zoological Nomenclature 58(4): 300.

229. Novokshonov VG, Rasnitsyn AP (2001) A new species of insects of the order Palaeomanteida = Miomoptera from the Lower Permian of Tshekarda (Perm Region). Vestnik Permskogo Universiteta, Geologiya [Bulletin of Perm’ University, Geology]3: 110–114 [In Russian].

230. Rasnitsyn AP (2002) The principles of taxonomy – phenetics, cladistics and traditional systematics. In: International Scientific Conference on the Systematics of Higher Plants, Dedicated to the 70th Anniversary of Associate Academician Prof. V.N. Tikhomirov: Abstracts (Moscow, 28–31 January, 2002). BCC Press, Moscow, 91–92 [In Russian].

231. Rasnitsyn AP (2002) Current state and agenda for paleoentomology. Vestnik Zoologii 36(1): 3–7 [In Russian].

232. Rasnitsyn AP, Quicke DLJ, editors (2002) History of Insects. Kluwer Academic Publishers, Dordrecht, xii+517 pp.

233. Rasnitsyn AP (2002) Scope and approach. In: Rasnitsyn AP, Quicke DLJ (Eds) History of Insects. Kluwer Academic Publishers, Dordrecht, 1–8 [total pages xii+517 pp.].

234. Rasnitsyn AP (2002) Special features of the study of fossil insects. In: Rasnitsyn AP, Quicke DLJ (Eds) History of Insects. Kluwer Academic Publishers, Dordrecht, 8–12 [total pages xii+517 pp.].

235. Rasnitsyn AP (2002) Concise history of palaeoentomology. In: Rasnitsyn AP, Quicke DLJ (Eds) History of Insects. Kluwer Academic Publishers, Dordrecht, 12–16 [total pages xii+517 pp.].

236. Rasnitsyn AP (2002) Class Insecta Linné, 1758. The insects (= Scarabaeoda Laicharting, 1781). In: Rasnitsyn AP, Quicke DLJ (Eds) History of Insects. Kluwer Academic Publishers, Dordrecht, 65–69 [total pages xii+517 pp.].

237. Rasnitsyn AP (2002) Subclass Lepismatona Latreille, 1804. The wingless insects (= Thysanura Latreille 1796, s.l.). In: Rasnitsyn AP, Quicke DLJ (Eds) History of Insects. Kluwer Academic Publishers, Dordrecht, 69–74 [total pages xii+517 pp.].

238. Rasnitsyn AP (2002) Subclass Scarabaeona Laicharting, 1781. The winged insects (= Pterygota Lang, 1888). In: Rasnitsyn AP, Quicke DLJ (Eds) History of Insects. Kluwer Academic Publishers, Dordrecht, 75–83 [total pages xii+517 pp.].

239. Rasnitsyn AP (2002) Infraclass Scarabaeones Laicharting, 1781. In: Rasnitsyn AP, Quicke DLJ (Eds) History of Insects. Kluwer Academic Publishers, Dordrecht, 84–85 [total pages xii+517 pp.].

240. Rasnitsyn AP (2002) Cohors Libelluliformes Laicharting, 1781 (= Subulicornes Latreille, 1807, = Hydropalaeoptera Rohdendorf, 1968). In: Rasnitsyn AP, Quicke DLJ (Eds) History of Insects. Kluwer Academic Publishers, Dordrecht, 85–89 [total pages xii+517 pp.].

241. Rasnitsyn AP, Pritykina LN (2002) Superorder Libellulidea Laicharting, 1781. Order Odonata Fabricius, 1792. The dragonflies. In: Rasnitsyn AP, Quicke DLJ (Eds) History of Insects. Kluwer Academic Publishers, Dordrecht, 97–104 [total pages xii+517 pp.].

242. Rasnitsyn AP (2002) Cohors Cimiciformes Laicharting, 1781. In: Rasnitsyn AP, Quicke DLJ (Eds) History of Insects. Kluwer Academic Publishers, Dordrecht, 104–115 [total pages xii+517 pp.].

243. Rasnitsyn AP (2002) Superorder Psocidea Leach, 1815. In: Rasnitsyn AP, Quicke DLJ (Eds) History of Insects. Kluwer Academic Publishers, Dordrecht, 125–133 [total pages xii+517 pp.].

244. Rasnitsyn AP (2002) Cohors Scarabaeiformes Laicharting, 1781. The holometabolans (= Holometabola Burmeister, 1835, = Endopterygota Sharp, 1899, = Oligoneoptera Martynov, 1938). In: Rasnitsyn AP, Quicke DLJ (Eds) History of Insects. Kluwer Academic Publishers, Dordrecht, 157–164 [total pages xii+517 pp.].

245. Rasnitsyn AP (2002) Order Jurinida M. Zalessky, 1928 (= Glosselytrodea Martynov, 1938). In: Rasnitsyn AP, Quicke DLJ (Eds) History of Insects. Kluwer Academic Publishers, Dordrecht, 189–192 [total pages xii+517 pp.].

246. Rasnitsyn AP (2002) Superorder Papilionidea Laicharting, 1781 (= Mecopteroidea Martynov, 1938). In: Rasnitsyn AP, Quicke DLJ (Eds) History of Insects. Kluwer Academic Publishers, Dordrecht, 192–194 [total pages xii+517 pp.].

247. Kozlov MV, Ivanov VD, Rasnitsyn AP (2002) Order Lepidoptera Linné, 1754. The butterflies and moths (= Papilionida Laicharting, 1781). In: Rasnitsyn AP, Quicke DLJ (Eds) History of Insects. Kluwer Academic Publishers, Dordrecht, 220–227 [total pages xii+517 pp.].

248. Rasnitsyn AP (2002) Order Pulicida Billbergh, 1820. The fleas (= Aphaniptera). In: Rasnitsyn AP, Quicke DLJ (Eds) History of Insects. Kluwer Academic Publishers, Dordrecht, 240–242 [total pages xii+517 pp.].

249. Rasnitsyn AP (2002) Superorder Vespidea Laicharting, 1781. Order Hymenoptera Linné, 1758 (= Vespida Laicharting, 1781). In: Rasnitsyn AP, Quicke DLJ (Eds) History of Insects. Kluwer Academic Publishers, Dordrecht, 242–254 [total pages xii+517 pp.].

250. Rasnitsyn AP (2002) Infraclass Gryllones Laicharting, 1781. The grylloneans (= Polyneoptera Martynov, 1938). In: Rasnitsyn AP, Quicke DLJ (Eds) History of Insects. Kluwer Academic Publishers, Dordrecht, 254–262 [total pages xii+517 pp.].

251. Vršanský P, Vishniakova VN, Rasnitsyn AP (2002) Order Blattida Latreille, 1810. The cockroaches (= Blattodea Brunner von Wattenvill [sic], 1882). In: Rasnitsyn AP, Quicke DLJ (Eds) History of Insects. Kluwer Academic Publishers, Dordrecht, 263–270 [total pages xii+517 pp.].

252. Rasnitsyn AP (2002) Superorder Perlidea Latreille, 1802 (= Plecopteroidea Martynov, 1938). In: Rasnitsyn AP, Quicke DLJ (Eds) History of Insects. Kluwer Academic Publishers, Dordrecht, 276–278 [total pages xii+517 pp.].

253. Rasnitsyn AP (2002) Order Embiida Burmeister, 1835. The webspinners (= Embioptera Shipley, 1904). In: Rasnitsyn AP, Quicke DLJ (Eds) History of Insects. Kluwer Academic Publishers, Dordrecht, 291–293 [total pages xii+517 pp.].

254. Gorochov AV, Rasnitsyn AP (2002) Superorder Gryllidea Laicharting, 1781 (= Orthopteroidea Handlirsch, 1903). In: Rasnitsyn AP, Quicke DLJ (Eds) History of Insects. Kluwer Academic Publishers, Dordrecht, 293–303 [total pages xii+517 pp.].

255. Rasnitsyn AP, Zherikhin VV (2002) Impression fossils. In: Rasnitsyn AP, Quicke DLJ (Eds) History of Insects. Kluwer Academic Publishers, Dordrecht, 437–444 [total pages xii+517 pp.].

256. Basibuyuk HH, Rasnitsyn AP, Fitton MG, Quicke DLJ (2002) The limits of the family Evaniidae (Insecta: Hymenoptera) and a new genus from Lebanese amber. Insect Systematics and Evolution 33(1): 23–34.

257. Zhang H, Rasnitsyn AP, Zhang J (2002) The oldest known scoliid wasps (Insecta, Hymenoptera, Scoliidae) from the Jehol biota of western Liaoning, China. Cretaceous Research 23(1): 77–86.

258. Zhang H, Rasnitsyn AP, Zhang J (2002) Pelecinid wasps (Insecta: Hymenoptera: Proctotrupoidea) from the Yixian Formation of western Liaoning, China. Cretaceous Research 23(1): 87–98.

259. Zherikhin VV, Rasnitsyn AP (2002) Second international paleoentomological congress. Paleontologicheskiy Zhurnal 2002(4): 112 [In Russian].

260. Rasnitsyn AP, Shcherbakov DE (2002) To the memory of Vladimir Vasilievich Zherikhin (1945–2001). Zoologicheskiy Zhurnal 81(6): 760–765 [In Russian].

261. Zhang H, Rasnitsyn AP, Zhang J (2002) Two ephialtitid wasps (Insecta, Hymenoptera, Ephialtitoidea) from the Yixian Formation of western Liaoning, China. Cretaceous Research 23(3): 401–407.

262. Rasnitsyn AP (2002) Evolutionary process and methodology of systematics. Trudy Russkogo Entomologicheskogo Obshchestva 73: 1–107 [In Russian].

263. Rasnitsyn AP, Sukacheva ID (2002) Permian and Triassic insects of the northeastern European Russia. In: Kruchinina NV, Krymgolts NG, Modzalevskaya TL (Eds) Problems of Biochronology in Paleontology and Geology. Abstracts of the XLIII Session of the Paleontological Society at the Russian Academy of Sciences. Russian Geological Institute, St. Petersburg, 114 [In Russian].

264. Dlussky GM, Rasnitsyn AP (2002 [2003]) Ants (Hymenoptera: Formicidae) of Formation Green River and some other Middle Eocene deposits of North America. Russian Entomological Journal 11(4): 411–436.

265. Rasnitsyn AP (2003) The present state and tasks of paleoentomology. Paleontologicheskiy Zhurnal 2003(2): 44–47 [In Russian].

266. Rasnitsyn AP, Ansorge J, Zessin W (2003) New hymenopterous insects (Insecta: Hymenoptera) from the Lower Toarcian (Lower Jurassic) of Germany. Neues Jahrbuch für Geologie und Paläontologie, Abhandlungen 227(3): 321–342.

267. Krassilov V, Tekleva M, Meyer-Melikyan N, Rasnitsyn A[P] (2003) New pollen morphotype from gut compression of a Cretaceous insect, and its bearing on palynomorphological evolution and palaeoecology. Cretaceous Research24(2): 149–156.

268. Zhang H, Rasnitsyn AP (2003) Some ichneumonids (Insecta, Hymenoptera, Ichneumonoidea) from the Upper Mesozoic of China and Mongolia. Cretaceous Research24(2): 193–202.

269. Rasnitsyn AP (2003) Obituary Viktor Grigorievich Novokshonov (26.02.1966–29.01.2003). Acta Zoologica Cracoviensia 46(Supplement – Fossil Insects): 11–15. [Krzemińska E, Krzemiński W (Eds), Proceedings of the 2nd Congress on Palaeoentomology “Fossil Insects”. Acta Zoologica Cracoviensia, 2003, 46(Supplement – Fossil Insects): 1–440].

270. Rasnitsyn AP (2003) On the skimming hypothesis of the origin ofinsect flight. Acta Zoologica Cracoviensia 46(Supplement – Fossil Insects): 85–88. [Krzemińska E, Krzemiński W (Eds), Proceedings of the 2nd Congress on Palaeoentomology “Fossil Insects”. Acta Zoologica Cracoviensia, 2003, 46(Supplement – Fossil Insects): 1–440].

271. Brothers DJ, Rasnitsyn AP (2003) Diversity of Hymenoptera and other insects in the Late Cretaceous (Turonian) deposits at Orapa, Botswana: a preliminary review. African Entomology 11(2): 221–226.

272. Rasnitsyn AP, Aristov DS, Gorokhov AV, Rowland JM, Sinitshenkova ND (2004) Important new insect fossils from Carrizo Arroyo and the Permo-Carboniferous faunal boundary. New Mexico Museum of Natural History and Science Bulletin 25: 215–246. [Lucas SG, Ziegler KE (Eds) Carboniferous-Permian Transition at Carrizo Arroyo, central New Mexico. New Mexico Museum of Natural History and Science Bulletin, 25, [iv]+1–300].

273. Rasnitsyn AP, Aristov DS (2004) Two new insects from the Upper Permian (Tatarian) of Belmont, New South Wales, Australia (Insecta: Hypoperlida: Anthracoptilidae = Permarrhaphidae; Grylloblattida: Sylvaphlebiidae). Paleontological Journal, 2004, 38(Supplement 2): S158–S163.

274. Sukacheva ID, Rasnitsyn AP (2004) Jurassic insects (Insecta) from the Sai-Sagul locality (Kyrgyzstan, southern Fergana). Paleontologicheskiy Zhurnal 2004(2): 64–68 [In Russian, with English translation in Paleontological Journal, 2004, 38(2): 182–186].

275. Rasnitsyn AP, Zhang H (2004) A new family, Daohugoidae fam. n., of siricomorph hymenopteran (Hymenoptera = Vespida) from the Middle Jurassic of Daohugou in Inner Mongolia (China). Trudy Russkogo Entomologicheskogo Obshchestva 75(1): 12–16.

276. Dlussky GM, Brothers DJ, Rasnitsyn AP (2004) The first Late Cretaceous ants (Hymenoptera: Formicidae) from southern Africa, with comments on the origin of the Myrmicinae. Insect Systematics and Evolution 35(1): 1–13.

277. Rasnitsyn AP, Basibuyuk HH, Quicke DLJ (2004) A basal chalcidoid (Insecta: Hymenoptera) from the earliest Cretaceous or latest Jurassic of Mongolia. Insect Systematics and Evolution 35(2): 123–135.

278. Rasnitsyn AP, Golovatch SI (2004) The identity of *Phryssonotus burmiticus* (Cockerell, 1917) (Diplopoda, Polyxenida, Synxenidae) in Cretaceous amber from Myanmar. Journal of Systematic Palaeontology 2(2): 153–157.

279. Rasnitsyn AP, Zhang H (2004) Composition and age of the Daohugou hymenopteran (Insecta, Hymenoptera = Vespida) assemblage from Inner Mongolia, China. Palaeontology47(6): 1507–1517.

280. Zhang J, Rasnitsyn AP (2004) Minute members of Baissinae (Insecta: Hymenoptera: Gasteruptiidae) from the Upper Mesozoic of China and limits of the genus *Manlaya* Rasnitsyn, 1980. Cretaceous Research 25(6): 797–805.

281. Zhang H, Rasnitsyn AP (2004) Pelecinid wasps (Insecta, Hymenoptera, Proctotrupoidea) from the Cretaceous of Russia and Mongolia. Cretaceous Research25(6): 807–825.

282. Rasnitsyn AP, Mostovski MB (2004) Viktor Grigorievich Novokshonov (26.02.1966–29.01.2003). Paleontological Journal38(Supplement 2): 75–79.

283. Rasnitsyn AP (2004) “Problemy Evolutsii [Problems of Evolution]. Vol. V. Edited by A.P. Kryukov and L.I. Yakimenko. Vladivostok, Dal’nauka. 2003. 304 p.” Zoologicheskiy Zhurnal 83(8): 1071–1072 [Book review; In Russian].

284. Rasnitsyn AP (2005) Selected Works on Evolutionary Biology. KMK Scientific Press, Moscow, Russia, iv+347 pp [In Russian] [Collection of earlier papers, except for: “Dynamics of taxonomic diversity: An afterword” from 2004, pp. 247–248].

285. Archibald SB, Rasnitsyn AP, Akhmetiev MA (2005) Ecology and distribution of Cenozoic Eomeropidae (Mecoptera), and a new species of *Eomerope *Cockerell from the Early Eocene McAbee locality, British Columbia, Canada. Annals of the Entomological Society of America 98(4): 503–514.

286. Rasnitsyn AP, Sukacheva ID, Aristov DS (2005) Permian insects of the Vorkuta Group in the Pechora Basin and their stratigraphic implications. Paleontologicheskiy Zhurnal 2005(4): 63–75 [In Russian, with English translation in Paleontological Journal, 2005, 39(4): 404–416].

287. Dlussky GM, Rasnitsyn AP (2005) Paleontological history of ants. In: Reznikova ZhI (Ed) Ants and Forest Protection: Materials of the 12th All-Russian Myrmecological Symposium, Novosibirsk, 7–14 August 2005. Novosibirsk State University, Novosibirsk, 49–53 [In Russian, with English summary].

288. Grimaldi D, Zhang J, Fraser NC, Rasnitsyn A[P] (2005) Revision of the bizarre Mesozoic scorpionflies in the Pseudopolycentropodidae (Mecopteroidea). Insect Systematics and Evolution36(4): 443–458.

289. Rinehart LF, Rasnitsyn AP, Lucas SG, Heckert AB (2005) Instar sizes and growth in the Middle Permian monuran *Dasyleptus brongniarti* (Insecta: Machilida: Dasyleptidae). New Mexico Museum of Science and History Bulletin 30: 270–272.

290. Rasnitsyn AP, Ansorge J, Zhang H (2006) Ancestry of the orussoid wasps, with description of three new genera and species of Karatavitidae (Hymenoptera = Vespida: Karatavitoidea stat. nov.). Insect Systematics and Evolution 37(2): 179–190.

291. Zhang H, Rasnitsyn AP (2006) Two new anaxyelid sawflies (Insecta, Hymenoptera, Siricoidea) from the Yixian Formation of western Liaoning, China. Cretaceous Research27(2): 279–284.

292. Rasnitsyn AP (2006) Fossil record and cladogram. In: Rozhnov SV (Ed) Evolution of the Biosphere and Biodiversity. KMK Scientific Press, Moscow, 39–48 [In Russian].

293. Rasnitsyn AP, Zhang H, Wang B (2006) Bizarre fossil insects: Web-spinning sawflies of the genus *Ferganolyda *(Vespida, Pamphilioidea) from the Middle Jurassic of Daohugou, Inner Mongolia, China. Palaeontology 49(4): 907–916.

294. Biryukova OB, Rasnitsyn AP (2006) Symbiotic relationships of ants with larvae of the sawfly family Blasticotomidae. In: CIS Symposium on Hymenoptera, Moscow, September 24–26, 2006: Program and Abstracts. Moscow State University, Moscow, 17 [total pages 98 pp.] [In Russian].

295. Rasnitsyn AP (2006) Early evolution of the higher Hymenoptera: New findings and new ideas. In: CIS Symposium on Hymenoptera, Moscow, September 24–26, 2006: Program and Abstracts. Moscow State University, Moscow, 74 [total pages 98 pp.] [In Russian].

296. Biryukova OB, Rasnitsyn AP, Novgorodova TA (2006) On trophobiotic interactions of ants with different insects. In: Entomological Research in North Asia. Proceedings of the VII Interregional Conference of Entomologists in Siberia and the Russian Far East, Novosibirsk, September 20–24, 2006]. Siberian Branch of the Russian Academy of Sciences, Novosibirsk, 203–205 [total pages 442 pp.] [In Russian].

297. Zhang J, Rasnitsyn AP (2006) New extinct taxa of Pelecinidae sensu lato (Hymenoptera: Proctotrupoidea) in the Laiyang Formation, Shandong, China. Cretaceous Research27(5): 684–688.

298. Rasnitsyn AP (2006) Classical and non-classical taxonomy: Another view. Zhurnal Obshchey Biologii 67(5): 385–388 [In Russian].

299. Agadjanian AK, Afanasjeva GA, Bannikov AF, Barskov IS, Bolshakova LN, Viskova LA, Vishnevskaya VS, Vorobjeva EI, Dzerzhinsky FY, Dmitrieva EL, Ivakhnenko MF, Krassilov AV, Kuzmina YM, Kurochkin EN, Leonova TB, Lopatin AV, Novikov IV, Novitskaya LI, Ponomarenko AG, Rasnitsyn AP, Rozhnov SV, Rozanov AY, Sokolov BS, Sukhanov VB, Tumanova TA, Fedonkin MA, Shishkin MA (2006) The birthday of Leonid Petrovich Tatarinov. Paleontologicheskiy Zhurnal 2006(6): 3–6 [In Russian, with English translation in Paleontological Journal, 2006, 40(6): 587–590].

300. Rasnitsyn AP (2006) Ontology of evolution and methodology of taxonomy. Paleontological Journal 40(Supplement 6): S679–S737.

301. Krassilov VA, Rasnitsyn AP, Afonin SA (2007) Pollen eaters and pollen morphology: Co-evolution through the Permian and Mesozoic. African Invertebrates 48(1): 3–11. [Brothers DJ, Mostovski MB, editors (2007) Congress Proceedings Fossils X3, Pretoria, South Africa, 7–11 February, 2005. African Invertebrates 48(1): [iv]+1–249].

302. Rasnitsyn AP, Brothers DJ (2007) Two new hymenopteran fossils from the mid-Cretaceous of southern Africa (Hymenoptera: Jurapriidae, Evaniidae). African Invertebrates 48(1): 193–202. [Brothers DJ, Mostovski MB, editors (2007) Congress Proceedings Fossils X3, Pretoria, South Africa, 7–11 February, 2005. African Invertebrates 48(1): [iv]+1–249].

303. Perkovsky EE, Rasnitsyn AP, Vlaskin AP, Taraschuk MV (2007) A comparative analysis of the Baltic and Rovno amber arthropod faunas: Representative samples. African Invertebrates 48(1): 229–245. [Brothers DJ, Mostovski MB, editors (2007) Congress Proceedings Fossils X3, Pretoria, South Africa, 7–11 February, 2005. African Invertebrates 48(1): [iv]+1–249].

304. Amitrov OV, Afanasjeva GA, Bannikov AF, Barskov IS, Bogoslovskaya MF, Boiko MS, Fedonkin MA, Gontsharova IA, Gorjunova RV, Iljina LB, Konovalova VA, Kuzina LF, Kuzmina YM, Kushlina VB, Lazarev SS, Leonova TB, Lopatin AV, Mazaev AV, Manankov IN, Mitta VV, Mikhailova IA, Nevesskaja LA, Nikolaeva SV, Novitskaya LI, Ponomarenko AG, Popov SV, Rasnitsyn AP, Rozhnov SV, Rozanov AY, Shishkin MA, Sokolov BS, Solovjev AN, Tatarinov LP, Viskova LA, Yanin BT, Yurina AL, Zhuravleva FA (2007) Aleksandr Aleksandrovich Shevyrev (1931–2006). Paleontologicheskiy Zhurnal 2007(3): 109–110 [In Russian, with English translation in Paleontological Journal, 2007, 41(3): 345–346].

305. Rasnitsyn AP (2007) On the discussion of the wing venation of (Archae)Orthoptera (Insecta). Paleontologicheskiy Zhurnal 2007(3): 105–108 [In Russian, with English translation in Paleontological Journal, 2007, 41(3): 341–344].

306. Brothers DJ, Rasnitsyn AP (2007) A new genus and species of Euparagiinae from the Late Cretaceous of South Africa (Hymenoptera: Vespidae). In: Abstract Book: FossilsX3, 4–9 May 2007. Diputación Foral de Álava, Vitoria-Gasteiz, 74 [total pages 275 pp.].

307. Rasnitsyn AP (2007) Hymenopterous insects in the Bembridge Marls (lowermost Oligocene, S. England) and in some other fossil sites. In: Abstract Book: FossilsX3, 4–9 May 2007. Diputación Foral de Álava, Vitoria-Gasteiz, 138 [total pages 275 pp.].

308. Rasnitsyn AP (2007) Origin and early diversification of the evanioid parasitic wasps. In: Abstract Book: FossilsX3, 4–9 May 2007. Diputación Foral de Álava, Vitoria-Gasteiz, 140 [total pages 275 pp.].

309. Zhang H, Rasnitsyn AP (2007) Nevaniinae subfam.n., a new subfamily of Praeaulacidae (Insecta: Hymenoptera: Evanioidea) from the Middle Jurassic of Daohugou in Inner Mongolia, China. In: Abstract Book: FossilsX3, 4–9 May 2007. Diputación Foral de Álava, Vitoria-Gasteiz, 186 [total pages 275 pp.].

310. Dlussky GM, Rasnitsyn AP (2007) Paleontological record and stages of ant evolution. Uspekhi Sovremennoy Biologii 127(2): 118–134 [In Russian].

311. Gokhman VE, Rasnitsyn AP (2006 [2007]) Symposium of CIS countries on hymenopterous insects. Russian Entomological Journal 15(4): 457–458 [In Russian].

312. Rasnitsyn AP, Gokhman VE, editors (2007) Studies on Hymenopterous Insects: Collection of Scientific Papers. KMK Scientific Press, Moscow, 263 pp. [In Russian].

313. Zhang H, Rasnitsyn AP, Wang D, Zhang Y (2007) Some hatchet wasps (Hymenoptera, Evaniidae) from the Yixian Formation of western Liaoning, China. Cretaceous Research28(2): 310–316.

314. Zhang H, Rasnitsyn AP (2007) Nevaniinae subfam. n., a new fossil taxon (Insecta: Hymenoptera: Evanioidea: Praeaulacidae) from the Middle Jurassic of Daohugou in Inner Mongolia, China. Insect Systematics and Evolution 38(2): 149–166.

315. Golubev VK, Shcherbakov DE, Rasnitsyn AP, Aristov DS, Sukacheva ID, Makarova OV, Silantyev VV (2007) Tikhie Gory. The Kazanian Stage fossil site of insects, fish, and plants. In: Geological Nature Monuments of the Tatarstan Republic. Aquarel-Art, Kazan’, 222–229 [In Russian].

316. Rasnitsyn AP (2007) Achievements, problems and prospects of palaeoentomology in the beginning of the XXI century. In: Zamotaylov AS (Ed) Prospects in General Entomology: Abstracts of the XIII Congress of the Russian Entomological Society, Krasnodar, 9–15 September 2007. Kuban’ State Agrarian University, Krasnodar, 306 [In Russian].

317. Rasnitsyn AP (2007) The problem of species revisited. Paleontological Journal 41(11): 1151–1155.

318. Vasilenko DV, Rasnitsyn AP (2007) Fossil ovipositions of dragonflies: Review and interpretation. Paleontological Journal41(11): 1156–1161.

319. Medvedev GS, Tobias VI, Emeljanov AF, Rasnitsyn AP, Richter VA, Kononova SV, Belokobylskii SA (2007) To the memory of M.A. Kozlov (1936–2006). Entomologicheskoe Obozrenie 86(2): 467–479 [In Russian, with English translation in Entomological Review, 2007, 87(5): 621–630].

320. Zherikhin VV, Ponomarenko AG, Rasnitsyn AP (2008) Introduction to Palaeoentomology. KMK Scientific Press, Moscow, 371 pp. [In Russian].

321. Sukacheva ID, Rasnitsyn AP (2008) Comparison of inclusion composition in extant and fossil resins. In: Bogdanova TN, Krymgolts NG (Eds) Geobiospheric Events and the History of the Organic World: These of the Reports of the LIV Session of the Palaeontological Society, Russian Academy of Science (7–11 April 2008, St. Petersburg). Russian Geological Research Institute, St. Petersburg, 170–172 [In Russian].

322. Ortega-Blanco J, Rasnitsyn AP, Delclòs X (2008) First record of anaxyelid woodwasps (Hymenoptera: Anaxyelidae) in Lower Cretaceous Spanish amber. Zootaxa1937: 39–50.

323. Krassilov V, Rasnitsyn A[P] (Eds) (2008) Plant-Arthropod Interactions in the Early Angiosperm History: Evidence from the Cretaceous of Israel. Brill, Leiden, 229 pp.

324. Anisyutkin LN, Grachev VG, Ponomarenko AG**, **Rasnitsyn AP, Vršanský P (2008) Part II: Fossil insects in the Cretaceous mangrove facies of southern Negev, Israel. In: Krassilov V, Rasnitsyn A[P] (Eds) Plant-Arthropod Interactions in the Early Angiosperm History: Evidence from the Cretaceous of Israel. Brill, Leiden, 189–223 [total pages 229 pp.].

325. Rasnitsyn AP (2008) New hymenopteran insects (Insecta: Vespida) from the Lower or Middle Jurassic of India. Paleontologicheskiy Zhurnal 2008(1): 84–87 [In Russian, with English translation in Paleontological Journal, 2008, 42(1): 81–85].

326. Ariunchimeg Y, Afanasjeva GA, Bogoslovskaya MF, Weiss OB, Vinogradov AV, Viskova LA, Vorob’eva EI, Gontar VI, Gorjunova RV, Grishchenko AV, Denisenko NV, Iljina LB, Lavrentjeva VD, Leonova TB, Lisitsyn DV, Lopatin AV, Manankov IN, Mezentseva OP, Nevesskaja LA, Nekhorosheva LV, Nikulina EA, Nikolaeva SV, Novikov IV, Ozhgibesov VP, Ostrovskii AN, Plamenskaya AG, Popeko LI, Pushkin VI, Rasnitsyn AP, Renga IO, Rozhnov SV, Roznova AY, Solovjev AN, Sukacheva ID, Schastlivtseva NP, Favorskaya TA, Ernst A (2008) Iraida Pavlovna Morozova (1919–2007). Paleontologicheskiy Zhurnal 2008(2): 110–112 [In Russian, with English translation in Paleontological Journal, 2008, 42(2): 218–220].

327. Zhang H, Rasnitsyn AP (2008) Middle Jurassic Praeaulacidae (Insecta: Hymenoptera: Evanioidea) of Inner Mongolia and Kazakhstan. Journal of Systematic Palaeontology 6(4): 463–487.

328. Brothers DJ, Rasnitsyn AP (2008) A new genus and species of Euparagiinae from the Late Cretaceous of southern Africa (Hymenoptera: Vespidae) Alavesia 2: 73–76.

329. Rasnitsyn AP (2008 [2009]) Hymenopterous insects (Insecta: Vespida) in the Upper Jurassic deposits of Shar Teg, SW Mongolia. Russian Entomological Journal 17(3): 299–310.

330. Aristov DS, Rasnitsyn AP (2008 [2009]) Position and taxonomy of the Permian fossil insect family Permembiidae (Insecta: Palaeomanteida = Miomoptera). Russian Entomological Journal 17(4): 327–334.

331. Rasnitsyn AP (2009) [untitled editorial in: Contributions to the discussion on electronic publication]. Bulletin of Zoological Nomenclature 66(1): 15–16.

332. Engel MS, Hinojosa-Díaz IA, Rasnitsyn AP (2009) A honey bee from the Miocene of Nevada and the biogeography of *Apis* (Hymenoptera: Apidae: Apini). Proceedings of the California Academy of Sciences, Series 4 60(3) 23–38.

333. Rasnitsyn AP, Brothers DJ (2009) New genera and species of Maimetshidae (Hymenoptera: Stephanoidea *s.l.*) from the Turonian of Botswana, with comments on the status of the family. African Invertebrates 50(1): 191–204.

334. Aristov DS, Wappler T, Rasnitsyn AP (2009) New and little known grylloblattids of the family Geinitziidae (Insecta: Grylloblattida) from the Triassic and Jurassic of Europe, Asia, and South Africa. Paleontologicheskiy Zhurnal 2009(4): 59–65 [In Russian, with English translation in Paleontological Journal, 2009, 43(4): 418–424].

335. Blank SM, Taeger A, Liston AD, Smith DR, Rasnitsyn AP, Shinohara A, Heidemaa M, Viitasaari M (2009) Studies toward a world catalog of Symphyta (Hymenoptera). Zootaxa2254: 1–96.

336. Ponomarenko AG, Rasnitsyn AP, Aristov DS, Lukashevich ED, Popov YA, Sinitshenkova ND, Sukacheva ID, Shcherbakov DE (2009) Jurassic Lagerstätte Shar-Teg. In: Paleontology of Central Asia. Abstracts of the International Conference on the 40th Anniversary of the Joint Russian-Mongolian Paleontological Expedition (JRMPE), November 18–19, 2009. Borissiak Paleontological Institute of the Russian Academy of Sciences, Moscow, 65–67 [In Russian].

337. Ren D, Labandeira CC, Santiago-Blay JA, Rasnitsyn AP, Shih C-K, Bashkuev A, Logan MAV, Hotton CL, Dilcher DA (2009) A probable pollination mode before angiosperms: Eurasian, long-proboscid scorpionflies. Science 326(5954): 840–847.

338. Sidorchuk EA, Rasnitsyn AP (2009) On the taxonomic position of *Palaeonothrus *Krivolutskii et Sidorchuk 2003 (Insecta: Hymenoptera: Ichneumonoidea, non Acariformes, Oribatida). Paleontologicheskiy Zhurnal 2009(6): 36–38 [In Russian, with English translation in Paleontological Journal, 2009, 43(6): 640–642].

339. Zherikhin VV, Sukacheva ID, Rasnitsyn AP (2009) Arthropods in contemporary and some fossil resins. Paleontological Journal 43(9): 987–1005.

340. Dlussky GM, Rasnitsyn AP (2009) Ants (Insecta: Vespida: Formicidae) in the Upper Eocene amber of central and eastern Europe. Paleontological Journal 43(9): 1024–1042.

341. Aristov DS, Rasnitsyn AP (2009 [2010]). The family Tillyardembiidae Zalessky, 1938 and the system of the plecopteroid insects. Russian Entomological Journal 18(4): 257–264.

342. Rasnitsyn AP (2010) Problems of molecular and morphological cladistics: A palaeontologist’s view. In: Timonin AC, Barykina RP, Zernov AS, Lotova LI, Novikov VS, Pimenov MG, Ploshinskaya ME, Sokoloff DD (Eds) XII Moscow Plant Phylogeny Symposium, Dedicated to the 250th Anniversary of Professor Georg Franz Hoffmann: Proceedings (Moscow, 2–7 February, 2010). KMK Scientific Press, Moscow, 59–62 [total pages 348 pp.] [In Russian].

343. Rasnitsyn AP, Aristov DS (2010) New Eoblattida (Insecta) from the Permian of Russia. Russian Entomological Journal 19(1): 13–20.

344. Rasnitsyn AP (2010) Molecular phylogenetics, morphological cladistics, and fossil record. Entomologicheskoe Obozrenie 89(1): 85–132 [In Russian, with English translation in Entomological Review, 2010, 90(3): 263–298].

345. Vasilenko DV, Rasnitsyn AP (2010) Fossil endophytic ovipositions. In: Problems of Aquatic Entomology of Russia and Adjacent Countries. Proceedings of the IV All-Russian Symposium on Amphibiotic and Aquatic Insects and X Trichopterological Symposium. North Osetian University Press, Vladikavkaz, 15–21 [in Russian].

346. Sukacheva ID, Rasnitsyn AP (2010) Inclusia in resins of contemporary trees as a clue to understanding of life in the amber forest. In: Amber Mining and Processing in Sambia: Abstracts of the International Symposium. Immanuel Kant Russian State University Press, Kaliningrad, 122–126 [in Russian].

347. Compton SG, Ball AD, Collinson ME, Hayes P, Rasnitsyn AP, Ross AJ (2010) Ancient fig wasps indicate at least 34 myr of stasis in their mutualism with fig trees. Biology Letters 6(6): 838–842.

348. Olmi M, Rasnitsyn AP, Guglielmino A (2010) Revision of rock fossils of Dryinidae and Embolemidae (Hymenoptera: Chrysidoidea). Zootaxa 2499: 21–38.

349. Ortega-Blanco J, Rasnitsyn AP, Delclòs X (2010) A new family of ceraphronoid wasps from Early Cretaceous Álava amber, Spain. Acta Palaeontologica Polonica 55(2): 265–276.

350. Gao T, Rasnitsyn AP, Ren D, Shih C (2010) The first Praesiricidae (Hymenoptera) from northeast China. Annales de la Société de Entomologique de France 46(1–2): 148–153. [Nel A, Petrulevičius JF, Azar D (Eds) Fossil Insects. Annales de la Société de Entomologique de France, 2010, 46(1–2): 1–295].

351. Brothers DJ, Rasnitsyn AP (2010) Upper Cretaceous (Turonian) Hymenoptera (Insecta) from Orapa, Botswana: An updated review. In: Proceedings of the 16th Conference of the Palaeontological Society of Southern Africa, Howick, August 5–8, 2010. Palaeontological Society of Southern Africa, Howick, 13.

352. Ortega-Blanco J, Delclòs X, Engel MS, McKellar RC, Peñalver E, Pérez-de la Fuente R, Perrichot V, Rasnitsyn AP, Soriano C (2010) Diversity of hymenopteran families in the Early Cretaceous amber from Spain. In: Program & Abstracts: FossilsX3, August 20–25, 2010. Capital Normal University, Beijing, 60.

353. Rasnitsyn AP, Zhang H (2010) Early evolution of Apocrita (Insecta, Hymenoptera) as indicated by new findings in the Middle Jurassic of Daohugou, NE China. In: Program & Abstracts: FossilsX3, August 20–25, 2010. Capital Normal University, Beijing, 89.

354. Perkovsky EE, Rasnitsyn AP, Vlaskin AP, Rasnitsyn SP (2010) Community structure in the Rovno amber forest (Late Eocene) as revealed by the study of the arthropod syninclusia. In: Program & Abstracts: FossilsX3, August 20–25, 2010. Capital Normal University, Beijing, 160.

355. Rasnitsyn AP, Zhang H (2010) Early evolution of Apocrita (Insecta, Hymenoptera) as indicated by new findings in the Middle Jurassic of Daohugou, northeast China. Acta Geologica Sinica84(4): 834–873.

356. Perkovsky EE, Rasnitsyn AP, Vlaskin AP, Rasnitsyn SP (2010) Community structure in the amber forest: Study of the arthropod syninclusia in the Rovno amber (Late Eocene of Ukraine). Acta Geologica Sinica84(4): 954–958.

357. Rasnitsyn AP (2010) Systems of the order Hymenoptera. In: II Symposium of the CIS on Hymenoptera (Insecta). St. Petersburg, 13–17 October, 2010. Zoological Institute, Russian Academy of Sciences, St. Petersburg, 120 [In Russian].

358. Aristov DS, Rasnitsyn AP (2010) Insects from the Upper Permian and basal Triassic of Angarida and Gondwana: A comparison. In: Golubev VK, Sennikov AG (Eds) Permian and Triassic Paleontology and Stratigraphy of North Eurasia: Materials of the 5th International Conference Devoted to the 150th Anniversary of Vladimir Prokhorovich Amalitzky (1860–1917), Moscow, 22–23 November 2010. Borissyak Paleontological Institute RAS [Russian Academy of Sciences], Moscow, 41–43 [In Russian].

359. Kopylov DS, Brothers DJ, Rasnitsyn AP (2010) Two new labenopimpline ichneumonids (Hymenoptera: Ichneumonidae) from the Upper Cretaceous of southern Africa. African Invertebrates 51(2): 423–430.

360. Olmi M, Rasnitsyn AP, Guglielmino A (2011) The first record of Embolemidae (Hymenoptera Chrysidoidea) in the Rovno Amber (Upper Eocene) of Ukraine: A male of *Ampulicomorpha succinalis* Brues. Paleontologicheskiy Zhurnal 2011(1): 66–68 [In Russian, with English translation in Paleontological Journal, 2011, 45(1): 73–76].

361. Rasnitsyn AP (2011) Arthropods in amber and other resins, both extant and extinct: Recent results obtained at the Paleontological Institute, Russian Academy of Sciences. In: Amberif 2011: 18th Seminar on Amber and its Zoological Inclusions, 12 March, 2011, Gdansk. Amberif, Gdansk, 7–14.

362. Aristov DS, Rasnitsyn AP (2011) A review of the family Protembiidae (Insecta: Eoblattida). Russian Entomological Journal 20(2): 119–127.

363. Rasnitsyn AP, van Dijk DE (2011) The first Gondwanan *Epimastax* from the Lopingian of KwaZulu-Natal, South Africa (Insecta: Palaeomanteida = Miomoptera: Permosialidae). African Invertebrates 52(1): 207–209.
